# Preoperative pembrolizumab (anti-PD-1 antibody) combined with chemoradiotherapy for esophageal squamous cell carcinoma: a phase 1/2 trial (PALACE-2)

**DOI:** 10.1038/s41392-025-02477-4

**Published:** 2025-11-28

**Authors:** Chengqiang Li, Yichao Han, Shengguang Zhao, Xiaozheng Kang, Yuyan Zheng, Yuqin Cao, Yan Yan, Liqiang Shi, Xipeng Wang, Tong Lu, Guowen Zou, Huan Li, Jiaming Che, Jie Xiang, Lianggang Zhu, Junbiao Hang, Yajie Zhang, Runsen Jin, Dingpei Han, Xueyu Chen, Hui Jing, Wei Guo, Zenghui Cheng, Liqin Zhao, Xiaoyan Chen, Bentong Yu, Jian Li, Bin Li, Yin Li, Hecheng Li

**Affiliations:** 1 Department of Thoracic Surgery, Ruijin Hospital, Shanghai Jiao Tong University School of Medicine, Shanghai, China; 2 Department of Radiotherapy, Ruijin Hospital, Shanghai Jiao Tong University School of Medicine, Shanghai, China; 3 Section of Esophageal and Mediastinal Oncology, Department of Thoracic Surgery, National Cancer Center/National Clinical Research Center for Cancer/Cancer Hospital, Chinese Academy of Medical Sciences and Peking Union Medical College, Beijing, China; 4 Department of Thoracic Surgery, The First Affiliated Hospital of Nanchang University, Jiangxi, China; 5 Department of Radiology, Ruijin Hospital, Shanghai Jiao Tong University School of Medicine, Shanghai, China; 6 Department of Oncology, Ruijin Hospital, Shanghai Jiao Tong University School of Medicine, Shanghai, China; 7 Department of Pathology, Ruijin Hospital, Shanghai Jiao Tong University School of Medicine, Shanghai, China; 8 Clinical Research Center, Ruijin Hospital, Shanghai Jiao Tong University School of Medicine, Shanghai, China; 9 Center for Immune-Related Diseases at Shanghai Institute of Immunology, Department of Respiratory and Critical Care Medicine of Ruijin Hospital, Department of Thoracic Surgery of Ruijin Hospital, Department of Immunology and Microbiology, Shanghai Jiao Tong University School of Medicine, Shanghai, China

**Keywords:** Gastrointestinal cancer, Tumour immunology, Cancer microenvironment, Cancer microenvironment

## Abstract

The role of preoperative anti-PD-1 antibody (pembrolizumab) plus chemoradiotherapy (PPCT) in locally advanced, resectable esophageal squamous cell carcinoma (ESCC) is still unclear. We aimed to investigate the therapeutic effect and safety of PPCT followed by surgery in this study (NCT03792347, NCT04435197). Patients with histologically confirmed, locally advanced, and surgically resectable ESCC were enrolled. They received PPCT with paclitaxel/carboplatin or nab-paclitaxel/carboplatin, followed by esophagectomy 4–6 weeks after treatment. The primary endpoint was the pathologic complete response (pCR) rate. Tumor specimens, blood samples and subcutaneous tumor mouse models were utilized to explore and validate the dynamic characteristics of the tumor microenvironment (TME) of ESCC after PPCT. Among the 143 patients enrolled, 140 received neoadjuvant treatment, and 125 underwent surgery. The pCR rate reached 43.2% (54/125). During neoadjuvant period, 75.7% (106/140) of patients experienced grade three or higher-grade adverse events. After a median follow-up of 17.4 months, patients showed a one-year disease-free survival rate of 91.1%, and an overall survival rate of 96.5%. Using scRNA-seq and cytokine profiling, we identified high IL-6 levels as a predictor of response to PPCT. In vivo experiment revealed that IL-6 neutralization enhanced the efficacy of immunotherapy by increasing CD4^+^ T-cell cytotoxicity. This is the first large-scale, multicenter, phase 1/2 trial reporting the short-term results of PPCT for locally advanced resectable ESCC. Although the short-term efficacy was not superior to that of neoadjuvant chemoradiotherapy, PPCT demonstrated acceptable safety and comparable one-year survival. We also revealed an association between the therapeutic response and the ability of anti-IL-6 blockade to enhance the efficacy of immunotherapy.

## Introduction

Esophageal carcinoma ranks as the 11th most prevalent malignancy and the 7th leading cause of cancer-related death globally.^1^ Esophageal squamous cell carcinoma (ESCC), a predominant subtype in developing regions, poses a particular challenge due to its aggressive nature and frequent late-stage diagnosis, with many patients first presenting with locally advanced disease.^2^ For these patients, neoadjuvant chemoradiotherapy (nCRT) followed by esophagectomy is the standard care.^3^

The landmark clinical trial, the Chemoradiotherapy for Esophageal Cancer Followed by Surgery Study (CROSS), demonstrated a pathologic complete response (pCR) rate of 49% after nCRT, accompanied by improved overall survival (OS) in ESCC.^4^ The NEOCRTEC5010 trial, conducted in a Chinese ESCC population, reached a pCR rate of 43.2% after nCRT.^5^ nCRT effectively controls over local recurrence postoperatively (decreasing from 34% to 14%). However, nCRT did not reduce the rate of distant recurrence, which emerged as the primary mode of relapse and constituted 70% to 80% of all recurrences.^6,7^ Consequently, a more efficacious strategy is needed to better control distant recurrence.

Immunotherapy induces the immune system to eliminate cancer and has the potential to become a significant approach for controlling distant metastasis.^8^ Specifically, immunotherapies targeting programmed cell death protein 1 (PD-1) or its ligand (PD-L1) have shown significant clinical effects on ESCC, as exemplified by the results of the KEYNOTE-590 and ESCORT-1st trials.^9,10^ In terms of neoadjuvant immunotherapy for resectable ESCC, the phase 3 ESCORT-NEO/NCCES01 trial^11^ revealed that the combination of camrelizumab with chemotherapy significantly improved the pCR rate (increased from 4.7% to 28%). Our previous PALACE-1 trial revealed that preoperative pembrolizumab in combination with chemoradiotherapy (PPCT) safely and feasibly induced a pCR rate of 55.6%.^12^ Reports on nCRT combined with immunotherapy are still limited to studies with small sample sizes,^12–14^ and there are currently no large-scale prospective studies.

We present here the short-term outcomes of the multicenter, single-arm, and phase 1/2 PALACE-2 trial, which represents the first prospective clinical trial with a relatively larger sample size to assess the effectiveness and safety of PPCT in locally advanced ESCC. Additionally, although many studies have applied single-cell sequencing to understand ESCC,^15–18^ research specifically focused on the response to PPCT remains limited. Therefore, we also performed an analysis of the dynamics of the tumor microenvironment (TME) in ESCC, which provided insights into the regulatory effects of our regimen.

## Results

### Baseline demographic and clinical characteristics

**Fig. 1:**
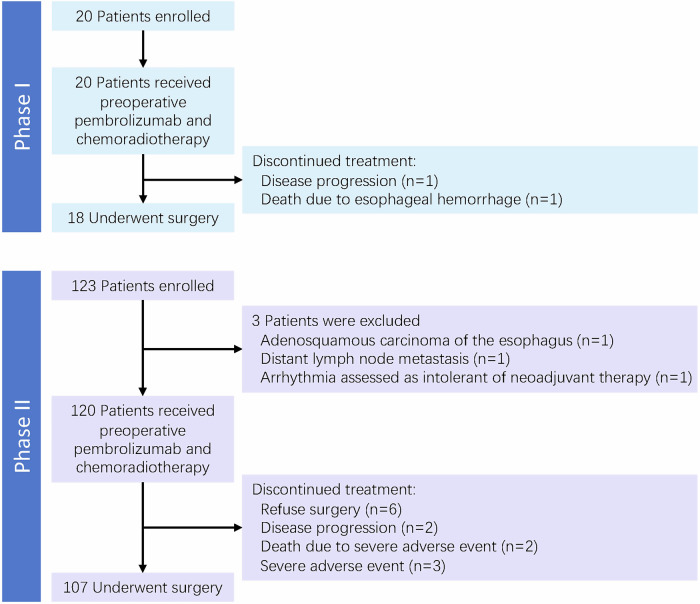
Flowchart of study enrollment

Between January 2019 and September 2023, 143 patients with locally advanced resectable ESCC were enrolled (Fig. 1). Three patients were excluded from the study: one due to adenosquamous carcinoma, another due to distant lymph node metastasis, and the third due to arrhythmia assessed as intolerant of neoadjuvant therapy during the baseline evaluation. Among the 140 patients who were included and received neoadjuvant therapy, 125 underwent curative esophagectomy.

The median age of the included patients was 63 years (interquartile range [IQR], 11 years). A majority of the participants were male (117/140, 83.6%), with females comprising 16.4% (23/140) of the study population (Supplementary Table 1). Among the enrolled patients, 99 (70.7%) had a history of smoking. ESCC was located in the distal third of the esophagus in 78 patients (55.7%), in the middle third in 51 patients (36.4%), and in the proximal third in 8 patients (5.7%). The median tumor length was 2.68 cm (IQR, 1.67 cm). Among the included cases of ESCC, 75 (52.1%) were classified as clinical stage III, 36 (25.7%) as stage II, and 31 (22.1%) as stage IVA.

### Surgical outcome

Among the 125 patients who underwent esophagectomy, McKeown surgery was performed for 81 patients (64.8%) and Ivor-Lewis surgery for 44 patients (35.2%) (Table 1). Most patients (97/125, 77.6%) received minimally invasive surgery. No patients required intraoperative conversion to open surgery. The R0 resection rate was 96.8% (121/125). The median number of removed lymph nodes was 18 (IQR, 15). Postoperative pathological staging revealed that 92 patients (73.6%) were classified as ypTNM stage I, seven patients (5.6%) as stage II, 18 patients (14.4%) as stage IIIA, and six patients (4.8%) as stage IIIB. A notable 43.2% (54/125;95% CI, 34.5–51.9%) of patients achieved pCR, with no observed association with postoperative PD-L1 combined positive score (CPS) (Supplementary Fig. 1). Furthermore, 48% (60/125) of patients attained a complete response of the primary tumor (tumor regression grade [TRG] 1), while 27.2% (34/125) achieved TRG 2. The overall major pathological response (mPR) rate for the primary tumor was 78.4% (98/125).

**Table 1:** Surgical and Pathological Outcomes (*n* = 125)

Characteristic	*n* (%)
**Surgical type**	
Ivor-Lewis	44 (35.2)
Mckeown	81 (64.8)
**Surgical technology**	
Thoracotomy	28 (22.4)
VATS	64 (51.2)
RATS	33 (26.4)
**Pathological T stage**	
pT0	59 (47.2)
pTis	1 (0.8)
pT1a	14 (11.2)
pT1b	15 (12.0)
pT2	21 (16.8)
pT3	14 (11.2)
pT4a	1 (0.8)
**Tumor regression grade**	
1	60 (48.0)
2	34 (27.2)
3	19 (15.2)
4	10 (8.0)
Not reported	2 (1.6)
**Pathological N stage**	
pN0	100 (80.0)
pN1	24 (19.2)
pN2	1 (0.8)
**Number of LNs dissected**	
Median	18
IQR	15
**Number of positive LNs**	
Median	0
Range	0–7
**Pathological TNM stage**	
I	92 (73.6)
II	7 (5.6)
IIIA	18 (14.4)
IIIB	6 (4.8)
IVA	2 (1.6)
**Major pathological response**	
Yes	98 (78.4)
No	21 (16.8)
Not reported	6 (4.8)
**Pathological complete response**	
Yes	54 (43.2)
No	71 (56.8)

*IQR* interquartile range, *LN* lymph node, *RATS* robotic-assisted thoracic surgery, *VATS* video-assisted thoracic surgery

### Safety and feasibility

Among the 140 patients who underwent PPCT, 28 (28/140, 20%) did not receive the full treatment regimen, mostly due to hematologic toxicity. Of these, 17 patients missed only one cycle of chemotherapy.

Most patients (139/140, 99.3%) experienced treatment-related adverse events (AEs). Grade three or higher AEs occurred in 75.7% of cases (106/140), with lymphopenia as the predominant AE (104/140, 74.3%), typically not necessitating additional intervention (Table 2). Others included anemia (4/140, 2.9%), immune-related pneumonitis (4/140, 2.9%), and neutropenia (2/140, 1.4%). Among the 140 patients, 125 proceeded to surgery. The reasons for not undergoing surgery primarily included patient refusal (*n* = 6), disease progression post-neoadjuvant therapy (*n* = 3), severe complications following neoadjuvant therapy (two pneumonitis and one toxic epidermal necrolysis), and patient deaths due to severe complications during neoadjuvant therapy (two esophageal hemorrhage and one immuno-related myocarditis, hepatitis, and pancreatitis). Among the patients who underwent surgery, the incidence of postoperative anastomotic leakage was 11.2% (14/125), while postoperative pneumonia occurred in 21.6% (27/125) of the patients. Additionally, 21 patients (16.8%) experienced hoarseness. One patient died within 90 days postoperatively due to severe esophagotracheal fistula.

**Table 2:** Adverse events during Neoadjuvant Pembrolizumab plus Chemoradiotherapy and after surgery

Events	*n* (%)
**Events of any grade during neoadjuvant therapy (***n* = **140)**	
Blood and lymphatic system disorders	
Lymphopenia	119 (85.0)
Leukopenia	101 (72.1)
Anemia	80 (57.1)
Thrombocytopenia	64 (45.7)
Decreased neutrophil count	47 (33.6)
Immune-related events	
Pneumonitis	4 (2.9)
Myocarditis	1 (0.7)
Hepatitis	1 (0.7)
Pancreatitis	1 (0.7)
Toxic epidermal necrolysis	1 (0.7)
Gastrointestinal disorders	
Nausea	19 (13.6)
Esophagitis	16 (11.4)
Vomiting	12 (8.6)
Constipation	8 (5.7)
Diarrhea	6 (4.3)
Esophageal hemorrhage	3 (2.1)
Skin and subcutaneous tissue disorders	
Alopecia	24 (17.1)
Rash	8 (5.7)
Dermatitis	7 (5.0)
General disorders	
Fatigue	22 (15.7)
**Events of grade** ≥ **3 during neoadjuvant therapy (***n* = **140)**	
Blood and lymphatic system disorders	
Lymphopenia	104 (74.3)
Leukopenia	4 (2.9)
Anemia	1 (0.7)
Thrombocytopenia	2 (1.4)
Decreased neutrophil count	2 (1.4)
Immune-related events	
Pneumonitis	4 (2.9)
Myocarditis	1 (0.7)
Hepatitis	1 (0.7)
Pancreatitis	1 (0.7)
Toxic epidermal necrolysis	1 (0.7)
Gastrointestinal disorders	
Esophageal hemorrhage	2 (1.4)
**Postoperative events (***n* = **125)**	
Pneumonia	27 (21.6)
Hoarseness	21 (16.8)
Anastomotic leakage	14 (11.2)
Chylothorax	4 (3.2)
Postoperative intrathoracic hemorrhage	2 (1.6)
Gastrointestinal fistula	1 (0.8)
Wound infection	1 (0.8)
Tracheoesophageal fistula	1 (0.8)
Cholecystitis	1 (0.8)

AEs were evaluated and recorded according to the CTCAE version 5.0

### Recurrence and survival

**Fig. 2:**
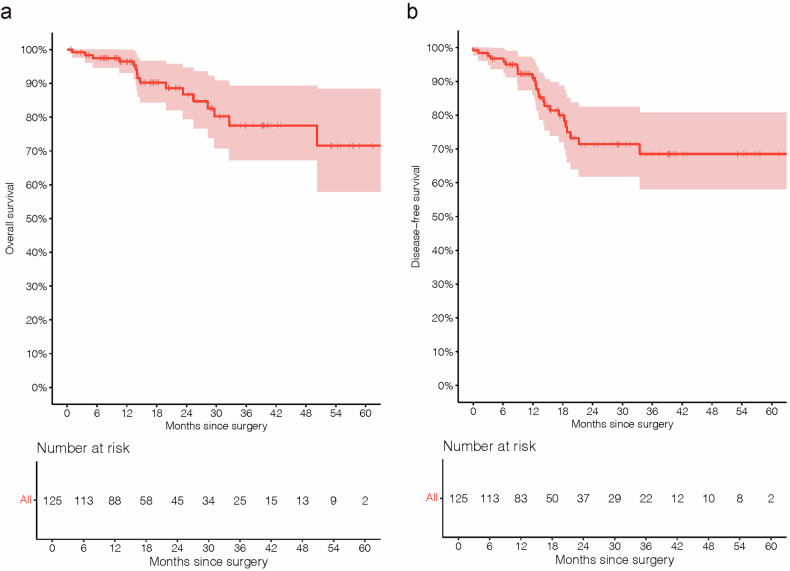
Kaplan-Meier curves for survival. **a** OS in the efficacy population. **b** DFS in the efficacy population. OS overall survival, DFS disease-free survival

With a median follow-up time of 17.4 months (range, 0.7–66.3 months), the median time to recurrence post-surgery in 22 patients was 12.6 months (range, 3.0-33.5 months). Tumor recurrence within one year and two years postoperatively occurred in nine patients (9/22, 40.9%) and 21 patients (21/22, 95.5%). The median DFS and OS were not reached at the time of data analysis. Patients who underwent surgery exhibited one- and two-year DFS rates of 91.1% and 71.4%, and OS rates of 96.5% and 86.7% (Fig. 2). The recurrence patterns of 22 patients with recurrence or metastasis are summarized in Supplementary Table 2. Locoregional recurrence was observed in 10 of 22 patients (45.5%), whereas 12 patients (12/22, 54.5%) experienced either distant or mixed recurrence.

### TME remodeling in ESCC after PPCT by single-cell RNA sequencing (scRNA-seq)

**Fig. 3:**
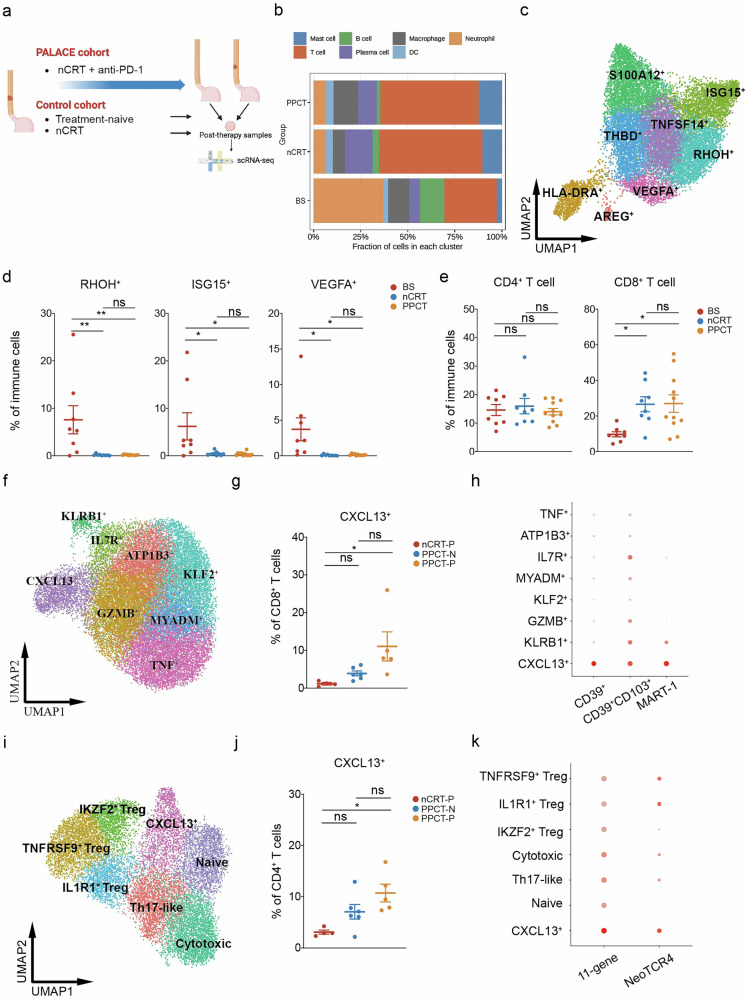
Preoperative pembrolizumab in combination with chemoradiotherapy modulates the tumor immune microenvironment. **a** Study design comparing PALACE cohort with control cohorts. **b** Stacked bar chart comparing the immune cell proportions among different groups. **c** UMAP plot highlighting neutrophil clusters. **d** Box plots comparing the proportions of RHOH^+^, ISG15^+^, and VEGFA^+^ neutrophils across different groups. **e** Box plots showing the proportions of CD4^+^ and CD8^+^ T cells across different groups. **f** UMAP plots showcasing distinct CD8^+^ T-cell clusters. **g** Box plot comparing the proportions of CXCL13^+^CD8^+^ T cells among different groups. **h** Dotplot displaying the expression of 3 signatures of previously reported tumor-reactive CD8^+^ T cells across all CD8^+^ T-cell clusters. **i** UMAP plots displaying distinct CD4^+^ T-cell clusters. **j** Box plot comparing the proportions of CXCL13^+^CD4^+^ T cells among different groups. **k** Dotplot showing the expression of 2 signatures of previously reported tumor-reactive CD4^+^ T cells across all CD4^+^ T-cell clusters. *P* values were derived from one-way ANOVA, Tukey’s test; ns: not significant, *: *P* < 0.05. Data are presented as mean ± SEM. *BS* treatment-naïve baseline, *nCRT* neoadjuvant chemoradiotherapy, PPCT preoperative pembrolizumab combined with chemoradiotherapy, PPCT-N non-pCR following PPCT, PPCT-P pCR following PPCT, pCR pathological complete response

To elucidate the alterations in the TME following PPCT in ESCC patients, we performed scRNA-seq on 11 tumor samples after PPCT (PPCT group). To distinguish the individual remodeling effects of neoadjuvant chemoradiotherapy and immunotherapy within the PPCT treatment, we also included two control cohorts: (i) 9 post-nCRT samples (nCRT group) and (ii) 8 treatment-naïve tumor samples served as baseline controls (BS group) (Fig. 3a; Supplementary Table 3). The clinical characteristics of these samples were similar across the groups (Supplementary Table 4). After stringent quality control and the exclusion of doublet cells, we analyzed a total of 169,183 cells. Following batch effect correction, we conducted unsupervised clustering, which revealed 12 distinct TME clusters, encompassing epithelial cells, fibroblasts, vascular smooth muscle cells (vSMCs), endothelial cells, neurons, and seven immune cell populations (Supplementary Fig. 2a). Each cluster was annotated based on the specific expression of canonical marker genes (Supplementary Fig. 2b).

To understand the dynamics of the TME after PPCT, we first examined the changes in major cell subsets. Both the nCRT and PPCT groups displayed a marked reduction in epithelial cells and an elevation in stromal cells (Supplementary Fig. 2c, d). The reduction in epithelial cells in these groups underscores the effectiveness of these neoadjuvant therapies in targeting and eliminating ESCC tumor cells. Conversely, stromal cells, including fibroblasts, endothelial cells, vSMCs, and neurons, showed significant or upward trends in their proportions in both the nCRT and PPCT groups, while immune cell populations remained consistent across the 3 groups.

### Increase in CD8^+^ T cells and reduction in neutrophil proportions after nCRT/PPCT

Given that nCRT can induce anti-tumor immunity, we conducted a detailed analysis of specific immune cell subsets. Firstly, myeloid cells were subdivided into macrophages, monocytes, neutrophils, conventional type 1 dendritic cells (cDC1s), conventional type 2 dendritic cells (cDC2s), mature dendritic cells enriched in immunoregulatory molecules (mregDCs), and cycling cells (Supplementary Fig. 3a). Of notice, neutrophil infiltration was significantly attenuated in both the nCRT and PPCT groups (Fig. 3b; Supplementary Fig. 3b). We further classified the neutrophil populations based on specific marker genes, identifying 8 distinct clusters (Fig. 3c; Supplementary Fig. 3c). Notably, Ras homolog family member H (RHOH)^+^, interferon-stimulated gene 15 (ISG15)^+^, and vascular endothelial growth factor A (VEGFA)^+^ neutrophils were the main subsets that were reduced following nCRT/PPCT (Fig. 3d; Supplementary Fig. 3d). ISG15^+^ neutrophils were reported to possess immunosuppressive capacity,^19^ while VEGFA^+^ neutrophils were recognized for their role in promoting angiogenesis.^20^ In contrast, RHOH^+^ neutrophils were not well characterized and may represent a subset with reduced effector function.^21^

The results showed that T-cell infiltration significantly increased following neoadjuvant chemoradiotherapy, with or without immunotherapy (nCRT/PPCT) (Fig. 3b; Supplementary Fig. 3e). T cells were further categorized based on canonical marker expression into CD4^+^ T cells, CD8^+^ T cells, natural killer (NK)/NKT cells, αβILTCK cells, γδT cells, innate lymphoid cells (ILCs), and cycling cells (Supplementary Fig. 3f, g). CD8^+^ T cells were the dominant T cell subset that significantly increased after nCRT/PPCT, indicating enhancement of the anti-tumor immune response (Fig. 3e). Additionally, γδT cells and ILCs also showed increases or a trend toward increased levels (Supplementary Fig. 3i). In addition, the proportions of other immune cell subsets were unaffected after nCRT/PPCT (Supplementary Fig. 4a).

### Expansion of tumor-reactive CD8^+^ T cells and CD4^+^ T cells driven by supplementary anti-PD-1 blockade

To assess the impact of anti-PD-1 blockade on T cells in addition to nCRT, we compared pCR samples (nCRT-P) from the nCRT group with both pCR samples (PPCT-P) and non-pCR samples (PPCT-N) within the PPCT group. The major T-cell subsets remained unchanged across the nCRT-P, PPCT-N, and PPCT-P groups (Supplementary Fig. 5a), prompting us to further analyze the subpopulations within CD4^+^ and CD8^+^ T cells. Based on gene expression profiles of CD8^+^ T cells, we identified two effector clusters (granzyme B [GZMB]^+^ and tumor necrosis factor [TNF]^+^), four memory clusters (Kruppel-like factor 2 [KLF2]^+^, interleukin-7 receptor [IL7R]^+^, ATPase Na^+^/K^+^ transporting subunit beta 1 [ATP1B]^+^, and myeloid-associated differentiation marker [MYADM]^+^), and one mucosal-associated invariant T (MAIT) cluster (killer cell lectin-like receptor B1 [KLRB1]^+^), as illustrated in the UMAP plot (Fig. 3f; Supplementary Fig. 5b). Interestingly, the number of C-X-C motif chemokine ligand 13 (CXCL13)^+^CD8^+^ T cells in the PPCT-P group significantly eclipsed that in the nCRT-P group, while the other CD8^+^ T-cell clusters remained comparable across the groups (Fig. 3g; Supplementary Fig. 5c). As described in several previous studies, CXCL13 expression distinguishes CD8^+^ T cells with tumor reactivity and reflects characteristics of exhaustion.^22,23^ We next assessed the tumor-reactive scores for each CD8^+^ T-cell subpopulation and verified that CXCL13^+^CD8^+^ T cells had the highest score (Fig. 3h; Supplementary Fig. 5d). Therefore, the observed increase in CXCL13^+^CD8^+^ T cells in the PPCT-P group suggests that anti-PD-1 blockade when combined with nCRT may drive the expansion of tumor-specific CD8^+^ T cells. We further analyzed the CD4^+^ T cells and identified 7 distinct clusters: naïve, T helper 17 (Th17)-like, cytotoxic, CXCL13^+^, and 3 regulatory T cell (Treg) subsets (Fig. 3i; Supplementary Fig. 5e). Notably, the level of CXCL13^+^CD4^+^ T cells was elevated in the PPCT-P group compared with the nCRT-P group (Fig. 3j). This subset is also thought to exhibit tumor-reactive characteristics, as evidenced by our finding showing a high tumor-reactive score, consistent with 2 previous studies (Fig. 3k; Supplementary Fig. 5f).^24,25^ These observations suggest that anti-PD-1 blockade elicits both tumor-specific CD8^+^ and CD4^+^ T-cell responses.

### Pathological regression under anti-PD-1 blockade correlated with robust cytotoxic programming and reduced immunosuppressive activity in CD4^+^ T cells, along with low serum interleukin-6 (IL-6) levels

To explore the underlying mechanisms of responsiveness to anti-PD-1 therapy in ESCC, we compared T-cell proportions between the PPCT-N and PPCT-P groups, using the nCRT-P group as a control. The proportion of CD8^+^ T-cell subsets showed no significant differences between the PPCT-N and PPCT-P groups. We compared CD8^+^ T-cell function using signature scores^26^ and found that activation/effector and cytotoxic functions were enhanced in the PPCT-P group (Supplementary Fig. 6a). Moreover, given that the CXCL13^+^ cluster exhibited features of exhaustion, we further analyzed and subdivided this cluster into precursor exhausted T cells (Tpex) and terminally exhausted T cells (Tex) (Supplementary Fig. 6b). However, both subpopulations were comparable between the two groups (Supplementary Fig. 6c).

**Fig. 4:**
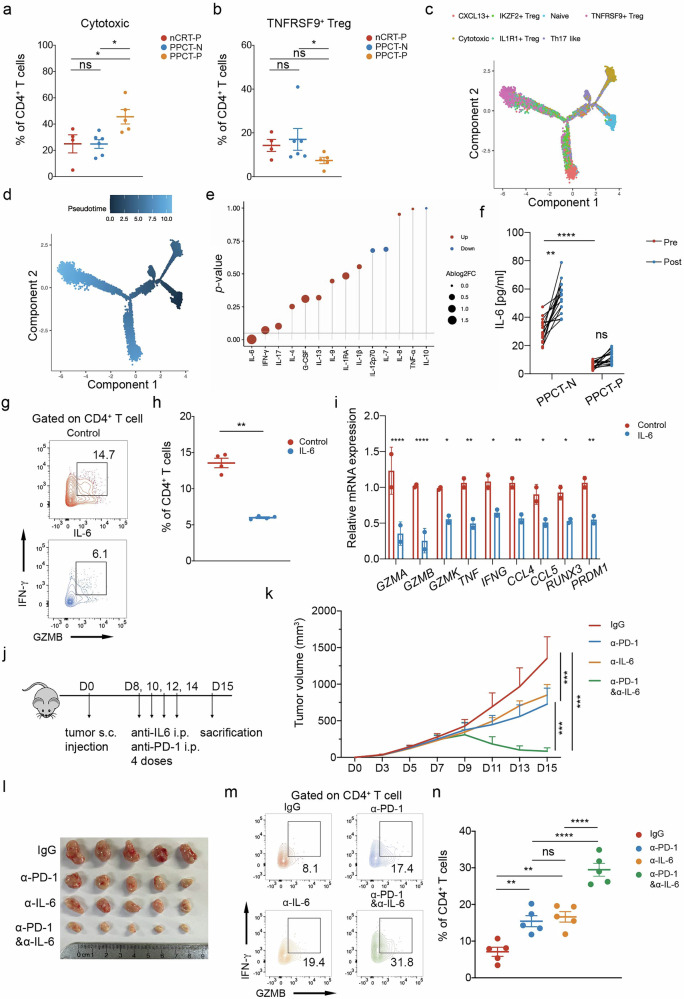
Pathological regression under anti-PD-1 blockade is associated with enhanced CD4^+^ T cell cytotoxic programming and reduced serum IL-6 levels. Box plot comparing the proportions of **a** cytotoxic CD4^+^ T cells and **b** TNFRSF9^+^ Tregs among different groups. **c** Branched trajectory of CD4^+^ T-cell state transition inferred by Monocle. **d** Trajectory plot showing the pseudotime curve of CD4^+^ T cells. **e** Comparison of baseline serum cytokine profiles in non-pCR samples compared to pCR samples following PPCT (*n* = 5 per group). **f** Serum IL-6 levels before and after PPCT, comparing the pCR and non-pCR groups (*n* = 16 matched pairs per group). **g** CD4^+^ T cells were activated, treated with IL-6, and analyzed for cytotoxicity after 24 hours. **h** Proportion of cytotoxic CD4^+^ T cells after IL-6 exposure. **i** RNA expression levels of effector genes, characteristic markers, and transcription factors associated with cytotoxic CD4^+^ T cells after IL-6 treatment. **j**–**n** LLC tumor-bearing mice were treated with anti-PD-1 and/or anti-IL-6 antibodies (*n* = 5 per group). **j** Schematic representation of the treatment protocol. **k** Tumor growth curves. **l** Final tumor sizes. **m** Tumor-infiltrating cytotoxic CD4^+^ T cell proportions. **n** Statistical comparison of cytotoxic CD4^+^ T cell levels. *P* values in (**a**, **b**, **k** and **n**) were derived from one-way ANOVA, Tukey’s test; *P* values in (**f**) were derived from 2-sided paired Student’s *t* test; *P* values in (**h**, **i**) were derived from 2-sided unpaired Student’s *t* test; ns: not significant, *P < 0.05, **P < 0.01, ***P < 0.001, ****P < 0.0001. Data are presented as mean ± SEM. BS treatment-naïve baseline; nCRT, neoadjuvant chemoradiotherapy; PPCT, preoperative pembrolizumab combined with chemoradiotherapy; PPCT-N, non-pCR following PPCT; PPCT-P, pCR following PPCT; pCR, pathological complete response

Subsequently, we conducted a detailed comparison of CD4^+^ T cells across the groups. Our analysis revealed a distinct subset of CD4^+^ T cells characterized by high expression of potent cytotoxic effector molecules, including granzyme A (GZMA), perforin-1 (PRF1), and interferon gamma (IFNγ) (Supplementary Fig. 6d). We designated this subset as cytotoxic CD4^+^ T cells, and their proportion was markedly elevated in the PPCT-P group compared with the PPCT-N group (Fig. 4a). In contrast, the overall frequency of Tregs was significantly reduced in the PPCT-P group (Supplementary Fig. 6e), with a particular decline in the tumor necrosis factor receptor superfamily member 9 (TNFRSF9)^+^ Treg subset (Fig. 4b), which is recognized for its robust immunosuppressive function.^27^

Then we conducted a pseudotime trajectory analysis of CD4^+^ cell clusters, which revealed that naïve CD4^+^ T cells can differentiate into cytotoxic CD4^+^ T cells or, alternatively, into other CD4^+^ T cells, predominantly Tregs at the opposite end of the spectrum (Fig. 4c). Notably, TNFRSF9^+^ Tregs appeared to represent the terminal stage of differentiation (Fig. 4d). To identify potential factors driving the transition of CD4^+^ T-cell states, we measured baseline serum cytokine levels using a cytokine protein chip. This analysis identified IL-6 as the only significantly downregulated cytokine in the PPCT-P group (Fig. 4e). To validate this finding, we expanded the sample size (16 matched pairs in each of the PPCT-P and PPCT-N groups) and confirmed the reduction in the baseline serum IL-6 concentration in the PPCT-P group using enzyme-linked immunosorbent assay (ELISA) (Fig. 4f). Additionally, we observed an increase in the post-treatment IL-6 level in the PPCT-N group that was not observed in the PPCT-P group. Elevated baseline serum IL-6 levels may predict unfavorable PPCT response, potentially by impairing the development of cytotoxic CD4^+^ T cells.

### IL-6 neutralization sensitizes anti-PD-1 blockade by augmenting CD4^+^ T cell cytotoxicity

We propose that IL-6 contributes to resistance to anti-PD-1 therapy by hindering the development of cytotoxic CD4^+^ T cells. To test this hypothesis, we assessed the expression of cytotoxic effector molecules by activated CD4^+^ T cells in vitro with and without IL-6 treatment. Notably, IL-6 treatment led to significant reductions in cytotoxic effector molecule expression levels (Fig. 4g, h). Furthermore, qPCR analysis revealed significant reductions in the expression of cytotoxic and effector-related genes in CD4^+^ T cells, as well as downregulation of transcription factors essential for cytotoxic CD4^+^ T-cell differentiation (Fig. 4i). To substantiate these findings, we employed a subcutaneous tumor model using a mouse ESCC cell line (mEC25) and treated the mice with anti-PD-1 monotherapy, anti-IL-6 monotherapy, or a combination of both (Fig. 4j). The combination therapy showed a superior therapeutic effect compared with either monotherapy, with tumors showing significant regression, underscoring the synergistic potential of these treatments (Fig. 4k, l). Notably, the proportion of CD4^+^ cytotoxic T lymphocytes showed the largest increase in the combination therapy group, highlighting a potential mechanism underlying the enhanced anti-tumor effect (Fig. 4m, n). The IFN-γ and GZMB expression levels in CD8^+^ T cells were highest in the combination therapy group, consistent with previous findings (Supplementary Fig. 7a, b).^28^ Moreover, as a well-known effect of IL-6, Th17 cell differentiation was attenuated by IL-6 blockade (Supplementary Fig. 7c, d). Thus, IL-6 neutralization with antibodies may serve as a viable therapeutic strategy to augment neoadjuvant immunotherapy in ESCC.

## Discussion

According to our literature review, this is the first large-scale, multicenter, prospective clinical trial focusing on PPCT for locally advanced resectable ESCC, which substantiates the safety and feasibility of this treatment model. At present, this study does not provide evidence that the pCR rate, the primary endpoint of PPCT, is superior to that of nCRT, and the 1-year and 2-year survival outcomes were similar to those of previous reports.^4,29^ Using tumor samples resected after PPCT, we conducted the first single-cell transcriptomic analysis exploring the impact of PPCT on the TME in ESCC.

Recently, immunotherapy has shown promising efficacy in advanced ESCC.^30–32^ As for the resectable ESCC, CheckMate-577 trial has demonstrated the considerable efficacy of adjuvant immunotherapy.^33^ In the phase 3 ESCORT-NEO/NCCES01 trial, the incorporation of immune checkpoint inhibitors (ICIs) resulted in a higher pCR rate compared with chemotherapy alone.^11^ The feasibility of combining nCRT with ICI was also evaluated for esophageal adenocarcinoma.^13^ As such, there is promising evidence that integration of ICIs can potentially enhance the clinical effectiveness of standard clinical regimens for resectable ESCC. We selected the CROSS regimen as the backbone for combining ICIs, given its status as a widely adopted standard nCRT approach for resectable ESCC,^4^ its use in previous nCRT–ICI trials,^13^, and its well-defined toxicity profile, in which the chemotherapy consisted of paclitaxel and carboplatin.^4^ In the phase II setting, paclitaxel was replaced with nab-paclitaxel, as supported by emerging evidence suggesting superior efficacy.^11,34,35^

In terms of our primary endpoint, the pCR rate was 43.2% (95% CI, 34.5% to 51.9%). Although the pCR rate observed in our study is higher than that reported in some nCRT studies,^36,37^ it appears to provide no additional benefit compared with the landmark CROSS and NEOCRTEC5010 trials.^4,5^ At a median follow-up time of 17 months, our trial exhibited comparable one- and two-year OS rates (96.5% and 86.7%, respectively) that were broadly comparable to those reported in the NEOCRTEC5010 trial (95% and 80%, respectively), cited here only as contextual references. And the impact of PPCT on long-term survival still requires further follow-up evaluation. We believe that future phase 3 trials should prioritize long-term survival endpoints rather than pCR.

The nCRT significantly reduces the postoperative local recurrence rate in ESCC, whereas no substantial improvement in the distant recurrence was observed.^7^ Immunotherapy offers potential to eliminate micro-metastatic tumor remnants that could precipitate distant recurrence.^38^ Preclinical investigations have also revealed synergistic interactions between radiotherapy and immunotherapy.^39,40^ Taken together, the PALACE regimen may yield better control of distant recurrence, but it requires further long-term follow-up data to be verified.

In terms of safety, similar to standard nCRT, hematologic toxicities were still the most common AEs.^4,5^ The most common grade 3 or higher AE was lymphopenia, which typically required no additional intervention. Our neoadjuvant treatment completion rate and surgical rate were comparable to those of standard CRT.^5^ Therefore, adding immunotherapy to nCRT resulted in no significant elevation in the risk of AEs, and PPCT demonstrated acceptable safety and feasibility. However, it should be noted that 3 patients were not scheduled for surgery due to treatment-related deaths. Among them, two were due to gastrointestinal bleeding, and one died from severe autoimmune hepatitis, pancreatitis, and myocarditis. One patient with gastrointestinal bleeding had marked baseline weight loss with a very low BMI (15.3), which may represent a pre-existing risk factor. Consequently, comprehensive pre-treatment evaluation and rigorous follow-up during therapy are crucial.

Recent studies have highlighted TME responses in ESCC to neoadjuvant therapy at single-cell resolution, focusing on CD8^+^ T cells, cDCs, and macrophages.^18,41,42^ Our data revealed an expansion of tumor-specific CD8^+^ and CD4^+^ T cells after PPCT treatment, suggesting that its therapeutic effect is likely mediated by T-cell responses. While the predictive value of baseline CXCL13^+^ cells requires further investigation, elevated levels of CXCL13^+^ CD8^+^ or CD4^+^ T cells may predict a favorable PPCT response, as indicated by recent findings from an ESCC neoadjuvant immunochemotherapy study.^43^ This trial offers the first single-cell transcriptomic analysis of PPCT’s impact on CD4^+^ T cells in ESCC TME. Our results revealed an increased cytotoxic CD4^+^ T-cell cluster in the PPCT-P group, contributing to the anti-tumor effects of anti-PD-1 therapy, consistent with findings that tumor-specific cytotoxic CD4^+^ T cells can kill autologous tumors and predict response to anti-PD-L1 therapy.^44^ Pre-treatment low serum IL-6 levels predicts anti-PD-1 efficacy across tumors,^45–47^ as IL-6–STAT3 signaling inhibits CD8^+^ T-cell cytotoxicity.^28^ Our study highlights cytotoxic CD4^+^ T cells as another IL-6-regulated subset impacting therapeutic outcomes. Moreover, IL-6 antibody neutralization may enhance the efficacy of neoadjuvant immunotherapy in ESCC.

Several limitations should be considered. Firstly, with its single-arm study design, data from a control group undergoing nCRT alone are lacking. To address this gap, we are currently conducting a prospective multicenter randomized controlled PALACE-3 trial (NCT06339060). Meanwhile, there are several ongoing phase 3 studies, similarly investigating nCRT for ESCC (NCT04973306, NCT04807673, NCT05357846, etc.). Secondly, the study population consisted only of Chinese patients, thus limiting the generalizability of the results.

In conclusion, we conducted the first large-scale, multicenter, single-arm, and phase I/II PALACE-2 trial. Although the pCR rate, our primary endpoint, was not superior to the previous reports, PPCT showed acceptable feasibility and safety, with the potential for long-term survival benefit that requires further investigation. Our study linked baseline IL-6 levels to immunotherapy efficacy and showed IL-6 blockade enhances anti-PD-1 therapy in ESCC.

## Materials and methods

### Study design and patients

The PALACE-2 trial is a multicenter, prospective, single-arm, and phase 1/2 clinical trial (ClinicalTrials.gov registration: NCT03792347 for phase 1, NCT04435197 for phase 2). Patients were recruited from three high-volume medical institutions in China: Ruijin Hospital, Shanghai Jiao Tong University School of Medicine; Cancer Hospital, Chinese Academy of Medical Sciences; and the First Affiliated Hospital of Nanchang University.

The histologically confirmed, locally advanced, and surgically resectable ESCC patients were enrolled. Locally advanced ESCC was defined as clinical stages ranging from cT2 to cT4a, with or without lymph node involvement, and no detectable metastatic spread. The primary endpoint was pCR rate, defined as the absence of residual cancer cells in surgically resected tissue samples. Secondary endpoints include the three-year disease-free survival (DFS) rate, three-year OS rate, R0 resection rate, and incidence of adverse events (AEs) during neoadjuvant therapy and the perioperative period.

The study protocol was approved by the Ethics Committees of the three participating institutions. All patients and their families provided written informed consent.

### PPCT and surgery

Chemotherapy consisted of intravenous carboplatin (area under the curve of two mg/mL/min) and paclitaxel/nab-paclitaxel (50 mg/m^2^), administered weekly for five weeks during the neoadjuvant treatment phase (on days 1, 8, 15, 22, and 29). Concurrent radiotherapy was delivered via external-beam radiation, with a total dose of 41.4 Gy in 23 fractions, with five fractions per week and 1.8 Gy per fraction. Pembrolizumab was co-administered on days one and 22 of the neoadjuvant therapy at a dosage of 200 mg. For patients who weighed less than 50 kg, the pembrolizumab dosage was adjusted to 100 mg. Esophagectomy was scheduled for four to six weeks after the completion of PPCT. The procedures included either open or minimally invasive esophagectomy (McKeown or Ivor-Lewis esophagectomy utilizing video-assisted or robotic-assisted techniques), along with a two-field or three-field lymphadenectomy.

Information on the detailed patient enrollment and the postoperative follow-up are documented in the Supplemental Materials.

### Pathological evaluation

For each case, the pathological stage was determined based on the criteria of the 8th edition of the American Joint Committee on Cancer for esophageal carcinoma,^48^ R0 resection is defined as a resection margin clear of tumor cells. According to the extent of remaining disease, the response of esophageal squamous cell carcinoma (ESCC) to preoperative pembrolizumab in combination with chemoradiotherapy (PPCT) was categorized into four groups according to the tumor regression grade from the College of American Pathologists^49^: grade 0, full response with no remaining cancer cells; grade 1, moderate response with isolated or small clusters of remaining cancer cells; grade 2, mild response marked by residual cancer clusters within significant interstitial fibrosis; and grade 3, no response with a substantial volume of remaining cancer cells and minimal to no necrosis of cancer cells. A major pathological response was defined as the presence of no more than 10% viable tumor cells in the primary lesions.^4,12^

### Clinical samples for TME evaluation

A total of 28 ESCC tumor samples were collected: 11 from the PALACE-2 trial (PPCT group), nine from patients with nCRT (nCRT group), and eight treatment-naïve tumor samples served as baseline controls (BS group). The nCRT group was subdivided into the nCRT-P group (pCR, *n* = 4) and the nCRT-N group (non-pCR, *n* = 5), while the PPCT group was divided into the PPCT-P group (pCR, *n* = 5) and the PPCT-N group (non-pCR, *n* = 6). Clinicopathological profiles are in Supplementary Table 3. The TME evaluation procedures are in Supplementary Materials.

### Statistical analysis

The per-protocol population was defined as all patients who received esophagectomy after PPCT. Perioperative AEs, short-term efficacy, and postoperative survival were analyzed in the per-protocol population. Patients receiving PPCT were included in the analysis for neoadjuvant toxicity. All information was documented and gathered through standardized case report forms for centralized analysis. We compared categorical variables with the Pearson Chi-squared test or Fisher's exact test. Normally distributed continuous variables were expressed as mean with standard deviation and compared using the Student t-test or one-way ANOVA test. Non-normally distributed continuous variables were displayed as median (interquartile range [IQR]) and compared using the Wilcoxon rank-sum test between groups. OS was calculated from surgery until death or last follow-up. Patients who were still alive at the last follow-up were right-censored. DFS was defined as the time from surgery to the first documented disease progression of local recurrence or distant metastasis, or death due to any cause. Patients who remained event-free at the last follow-up were right-censored. OS and DFS at one and two years were calculated using the Kaplan–Meier method. The statistical details were indicated in the legends. Statistical analyses employed conventional two-tailed tests with a significance level set at α = 0.05 and two-sided 95% confidence intervals. *P* < 0.05 was statistically significant (*P < 0.05, **P < 0.01, ***P < 0.001, ****P < 0.0001). Bars in graphs represent mean ± standard error of mean. GraphPad Prism (version 8·0, GraphPad Software Inc., La Jolla, CA, USA), R (version 4.1.2, R Foundation for Statistical Computing, Vienna, Austria), and IBM SPSS Statistics (version 22, IBM Inc., Armonk, USA) were utilized for analyses.

## Supplementary information

Supplementary information

Supplementary information

Supplementary information

## Data Availability

The raw scRNA-seq data generated in this study have been deposited in the Genome Sequence Archive (GSA) under accession number HRA012626 (https://ngdc.cncb.ac.cn/gsa-human/browse/HRA012626).
