# Supplementary material for: Preoperative pembrolizumab (anti-PD-1 antibody) combined with chemoradiotherapy for esophageal squamous cell carcinoma: a phase 1/2 trial (PALACE-2)
Source: Signal Transduct Target Ther. 2025 Nov 28;10:386. doi: 10.1038/s41392-025-02477-4 (PMC12660869; doi:10.1038/s41392-025-02477-4)
Supplement: Supplementary file 1 — Supplementary information [file 41392_2025_2477_MOESM1_ESM.docx]

Supplementary Materials for

Preoperative pembrolizumab (anti-PD-1 antibody) combined with chemoradiotherapy for esophageal squamous cell carcinoma: a phase 1/2 trial (PALACE-2)

Chengqiang Li, Yichao Han, Shengguang Zhao, Xiaozheng Kang, Yuyan Zheng, Yuqin Cao, Yan Yan, Liqiang Shi, Xipeng Wang, Tong Lu, Guowen Zou, Huan Li, Jiaming Che, Jie Xiang, Lianggang Zhu, Junbiao Hang, Yajie Zhang, Runsen Jin, Dingpei Han, Xueyu Chen, Hui Jing, Wei Guo, Zenghui Cheng, Liqin Zhao, Xiaoyan Chen, Bentong Yu, Jian Li, Bin Li, Yin Li, Hecheng Li

Correspondence to: lihecheng2000@hotmail.com

**This PDF file includes:**

Materials and Methods

Supplementary Text

Figures. S1 to S7

Tables S1 to S4

**Other Supplementary Materials for this manuscript include the following:**

Study Protocol

Statistical Analysis Plan

Materials and Methods

Patient enrollment

This study enrolled patients with histologically confirmed, locally advanced, and surgically resectable ESCC. The presence of positive PD-L1 expression was not a requirement for inclusion. Locally advanced ESCC was defined as clinical stages ranging from cT2 to cT4a, with or without lymph node involvement, and no detectable metastatic spread. Enrolled patients ranged in age from 18 to 75 years. Patients were excluded if they had: a history of any autoimmune disorder, need for systemic treatment with corticosteroids or other immunosuppressive medications, any previous malignancy, or symptomatic interstitial pulmonary disease.

Management of adverse events (AEs)

During the neoadjuvant phase, management of AEs related to chemoradiotherapy involved clinical monitoring, dose adjustments, temporary cessation of chemotherapy or radiotherapy, and administration of symptomatic treatment according to the specific type and severity of AEs,^1, 2^ under guidance from multidisciplinary consultations. Modification of the pembrolizumab dosage was not advised for immune-related AEs.

Patient follow-up

Postoperative follow-up was arranged according to the guidelines provided by the National Comprehensive Cancer Network for esophageal cancer,^3^ with follow-up visits at 1, 6, 12, 18, 24, 30, 36, 48, and 60 months post-surgery. For patients who exhibited signs of potential local recurrence or distant metastasis, additional visits were scheduled, and contrast-enhanced neck, chest, and abdominal computed tomography, positron emission tomography-computed tomography, and/or upper gastrointestinal endoscopy were conducted.

Sample size estimation

Based on previously published data, the anticipated pathological complete response (pCR) rate following neoadjuvant chemoradiotherapy (nCRT) for locally advanced ESCC in the Asian population was projected to be 43·2%.^4^ In contrast, from the preliminary findings of the PALACE-1 study, the expected pCR rate after PPCT was estimated to be 56%.^5^ To achieve 80% power and a 5% significance level for detecting a difference in the pCR rates between PPCT and chemoradiotherapy, a required sample size of 130 was calculated. Considering a 10% dropout rate, the sample size was adjusted to 143 for the PALACE-2 trial.

Sample preparation

ESCC tumors were immediately dissected following specimen collection, preserved in tissue storage solution (Miltenyi Biotec, Germany), and stored at a temperature of 2–8°C until processing. Briefly, tumor samples were washed with 1×PBS, then minced into small pieces (~1mm^3^) on ice. The tissue was enzymatically digested using a Tumor Dissociation Kit (Miltenyi Biotec, Germany) and one mg/mL Dispase II (Worthington, USA) for 35 min at 37°C with continuous agitation. Cells were collected by centrifugation at 350 g for ten min, washed, filtered through a 35 μm cell strainer, and resuspended in ice-cold PBS. To assess cell viability, samples were stained with Calcein-AM (Thermo Fisher Scientific, USA) and Draq7 (BD Biosciences, USA) were used for staining. Finally, the single-cell suspension was enriched by removing dead cells using the MACS Dead Cell Removal Kit (Miltenyi Biotec).

single-cell RNA sequencing (scRNA-seq)

The BD Rhapsody system was utilized to capture transcriptomic data from individual cells. Approximately 8,000 cells were distributed randomly across more than 200,000 microwells via limited dilution, to allow for single-cell capture. Oligonucleotide barcode-laden beads were added to ensure saturation, with each microwell containing one bead paired with a single cell. Upon cell lysis, mRNA molecules hybridized with the barcoded capture oligos on the beads. The beads were then pooled for reverse transcription and ExoI digestion. During cDNA synthesis, each cDNA molecule was tagged at the 5’ end with a unique molecular identifier (UMI) and a cell-specific barcode. Whole transcriptome libraries were constructed using the BD Rhapsody single-cell whole-transcriptome amplification workflow. The libraries were quantified using a High Sensitivity DNA chip (Agilent, USA) on a Bioanalyzer 2200 with a Qubit High Sensitivity DNA assay (Thermo Fisher Scientific, USA). Sequencing was performed on the Illumina NovaSeq6000 platform (Illumina, USA) using a 150 bp paired-end run.

scRNA-seq data analysis

scRNA-seq data were processed using Fastp with default settings to generate clean reads, and the cell barcode whitelist was identified using UMI-tools. The UMI-filtered clean reads were then mapped to the human genome (Ensembl version 100) using STAR, with customized parameters from the UMI-tools standard pipeline, to obtain UMI counts for each sample. The quality filtering criteria for cells were the expression of more than 200 genes and a mitochondrial UMI rate of less than 20%.

The Seurat package (version 4·1·0) was used for most of the scRNA-seq analysis. Variable genes were detected using the ‘FindVariableFeatures’ function, and then the expression data were scaled with the ‘ScaleData’ function. Principal components (PCs) were computed using the ‘RunPCA’ function. Batch effect was adjusted using the Harmony package (version 0·1·1), after clustering with the ‘FindNeighbors’ and ‘FindClusters’ functions based on the top PCs. Dimensionality reduction was performed using UMAP and tSNE, calculated with the ‘RunUMAP’ and ‘RunTSNE’ functions, respectively. Cell clusters were identified and visualized using the ‘DimPlot’ function, based on the coordinates of UMAP. Marker genes were identified with the ‘FindMarkers’ function and visualized using the ‘FeaturePlot’ and ‘DotPlot’ functions. Tumor-reactive T-cell signature scoring was conducted using the ‘AddModuleScore’ function in Seurat with default parameters. We applied the Monocle (version 2·26·0) algorithm with the genes of CD4^+^ T-cell clusters as input to determine the differentiation trajectory.

Pathway analysis

The ‘FindMarkers’ function in Seurat was employed to calculate the P value and log2 (fold change) (log2 FC) between two clusters. Additionally, a preranked gene set enrichment analysis was conducted using ‘clusterProfiler’ (version 4·6·2), with gene ranking based on the log2 FC of CD8^+^/CD4^+^ cell clusters.

Cytokine detection

For the detection of cytokine expression, the Luminex liquid suspension chip was employed along with the Bio-Plex Pro Human Cytokine Panel 27-plex kit, following the manufacturer’s protocol (Bio-Rad, USA). Conditioned media samples from each group, with three replicates, were incubated in 96-well plates containing microbeads for one hour. Subsequently, detection antibodies were added and incubated for 30 min. Streptavidin-phycoerythrin (PE) was then introduced to each well for ten min. The results were measured using the Bio-Plex MAGPIX System (Bio-Rad). Human interleukin-6 (IL-6) levels were measured using a Human IL-6 enzyme-linked immunosorbent assay Kit (Invitrogen, USA), following the manufacturer’s protocol.

Murine tumor studies

For the murine tumor model, mEC25 tumor cells (5×10^6^) were subcutaneously engrafted into the right flanks of age-matched, 6–8-week-old male C57BL/6J mice. Tumor volume was calculated using the formula: Volume = 1/2 (Length×Width^2^), based on measurements taken with digital calipers. Mice bearing mEC25 tumors were randomly divided into four groups of five mice each and received intraperitoneal injections of four different panels of antibodies dissolved in 200 µl PBS (1×, pH 7·4) on days 8, 10, 12, and 14. The four panels of antibodies were: isotype control antibodies, anti- programmed cell death protein 1 (PD-1) (rat IgG2a clone RMP1-14, 10 mg/kg), anti-IL-6 (rat IgG1 clone MP5-20F3, 10 mg/kg), and anti-PD-1 (10 mg/kg) plus anti-IL-6 (10 mg/kg). Rat IgG2a (clone 2A3, 10 mg/kg) and rat IgG1 (clone HRPN, 10 mg/kg) were used as isotype control antibodies. Mice were euthanized on day 15, following a protocol approved by the Institutional Animal Care and Use Committee of Ruijin Hospital (IACUC: RJ2024020). Tumors were dissociated by mechanical mincing followed by incubation in solution containing one mg/mL Collagenase IV (Sigma-Aldrich, USA) and 150 μg/mL DNase I (Sigma-Aldrich).

CD4^+^ T cell isolation, culture, and activation

Peripheral blood mononuclear cells (PBMCs) were isolated from a healthy donor via Ficoll Paque Plus (GE Healthcare, USA) density gradient centrifugation. CD4^+^ T cells were then purified from PBMCs using anti-human CD4 MACS beads (Miltenyi Biotec) following the manufacturer’s protocol. The isolated CD4^+^ T cells were seeded at 150,000 cells per well in a 96-well plate and stimulated with three µg/mL plate-bound anti-CD3 (InVivoMAb, USA) and two µg/mL soluble anti-CD28 (InVivoMAb) in 200 µL culture medium (RMPI 1640 supplemented with 10% fetal bovine serum, 1% penicillin/streptomycin, and 50 mM 2-mercaptoethanol) supplemented with 50 U/mL recombinant human IL-2 (R&D Systems, USA). After 48 h of stimulation, the activated CD4^+^ T cells were treated with recombinant human IL-6 (Peprotech, USA) for 24 h, after which they were collected for further analysis.

Flow cytometric analysis

Single-cell suspensions from tumor tissue samples were washed and incubated with a Live/Dead dye kit (Fixable Viability, eBioscience, USA) in PBS for 15 min at 4°C. Following incubation, the cells were washed in fluorescence-activated cell sorting (FACS) buffer (PBS containing 2% fetal bovine serum and 2 mM EDTA). Fc block and antibodies were diluted in FACS buffer at the appropriate concentrations. Cells were stained with the designated surface antibodies for 30 min at 4°C before being washed and resuspended in FACS buffer. The surface antibodies included PerCP/Cy5·5 anti-human CD4 (300530, BioLegend, USA, 1:200), PerCP-Cy5·5 anti-mouse T-cell receptor beta (109227, BioLegend, 1:300), PE-Cy7 anti-mouse CD4 (100527, BioLegend, 1:200), and BV421 anti-mouse CD8a (563898, BD Pharmingen, 1:300). For intracellular staining, cells were fixed, permeabilized, and labeled with antibodies such as fluorescein isothiocyanate-labeled anti-human/mouse granzyme B (GZMB) (372206, BioLegend, 1:200), PE-labeled anti-human interferon gamma (IFNγ) (502510, BioLegend, 1:200), PE-labeled anti-mouse IFNγ (12-7311-82, eBioscience, 1:200), and PE-labeled anti-mouse IL-17A (130-102-344, Miltenyi Biotec, 1:200). To detect cytokine expression, cells were stimulated with phorbol 12-myristate 13-acetate (Sigma-Aldrich), ionomycin (Sigma-Aldrich), and GolgiStop (BD Bioscience, USA) for 4–6 h prior to staining.

References

1. van Hagen, P. *et al.* Preoperative chemoradiotherapy for esophageal or junctional cancer. *N Engl J Med* **366**, 2074-2084 (2012).

2. Kojima, T. *et al.* Randomized Phase III KEYNOTE-181 Study of Pembrolizumab Versus Chemotherapy in Advanced Esophageal Cancer. *J Clin Oncol*, Jco2001888 (2020).

3. Rice, T.W., Ishwaran, H., Ferguson, M.K., Blackstone, E.H. & Goldstraw, P. Cancer of the Esophagus and Esophagogastric Junction: An Eighth Edition Staging Primer. *J Thorac Oncol* **12**, 36-42 (2017).

4. Ajani, J.A. *et al.* Esophageal and Esophagogastric Junction Cancers, Version 2.2019, NCCN Clinical Practice Guidelines in Oncology. *J Natl Compr Canc Netw* **17**, 855-883 (2019).

5. Li, C. *et al.* Preoperative pembrolizumab combined with chemoradiotherapy for oesophageal squamous cell carcinoma (PALACE-1). *Eur J Cancer* **144**, 232-241 (2021).

6. Ajani, J.A. *et al.* Esophageal and Esophagogastric Junction Cancers, Version 2.2023, NCCN Clinical Practice Guidelines in Oncology. *J Natl Compr Canc Netw* **21**, 393-422 (2023).

7. Yang, H. *et al.* Neoadjuvant Chemoradiotherapy Followed by Surgery Versus Surgery Alone for Locally Advanced Squamous Cell Carcinoma of the Esophagus (NEOCRTEC5010): A Phase III Multicenter, Randomized, Open-Label Clinical Trial. *J Clin Oncol* **36**, 2796-2803 (2018).

**
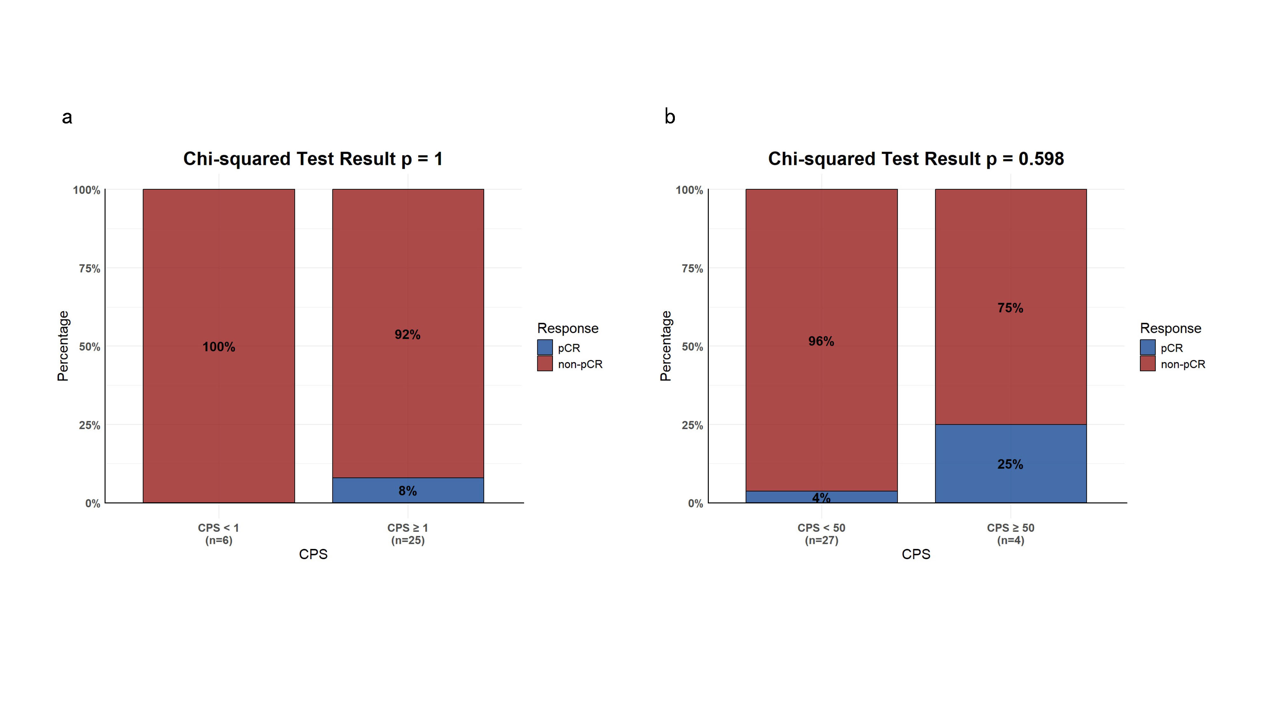
**

Supplementary Fig. 1. The association of pCR and postoperative PD-L1 combined positive score (CPS) by stratifying patients into high- and low- CPS groups.

(a) The proportion of patients achieving pCR between the high- and low- CPS groups, using cutoff values of 1.

(b) The proportion of patients achieving pCR between the high- and low- CPS groups, using cutoff values of 50.

**
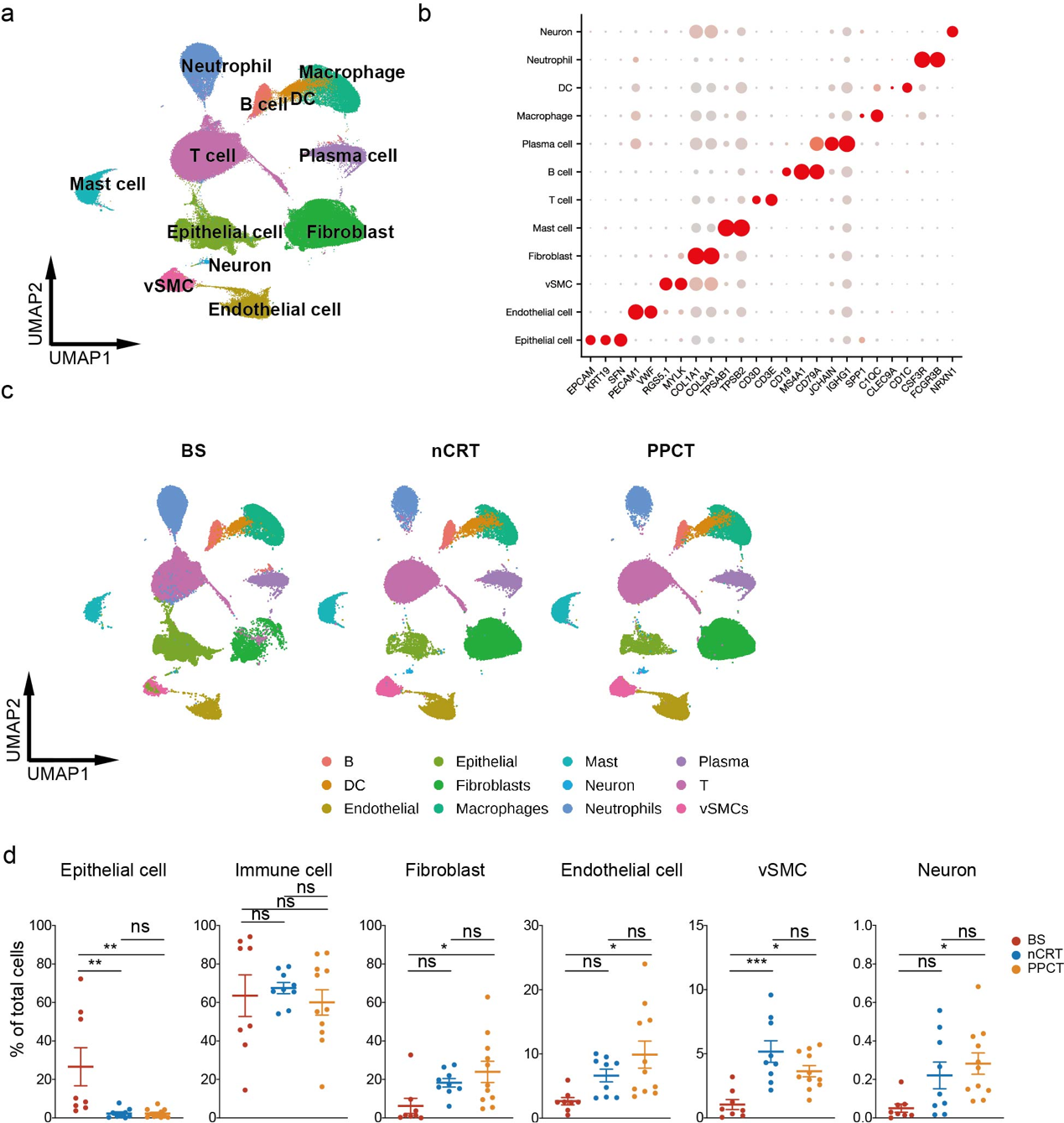
**

Supplementary Fig. 2. Major cell types in the tumor microenvironment of ESCC.

(a) UMAP plot showing major cell types. (b) Dotplot illustrating the expression levels of key marker genes that define each major cell type. (c) UMAP plots comparing the distribution of major cell types across different groups. (d) Box plots displaying the proportion of epithelial cells, immune cells, fibroblasts, endothelial cells, vSMCs and neuron among different groups. *P* values were derived from one-way ANOVA, Tukey’s test; ns: not significant, *: *P* < 0.05, **: *P* < 0.01, ***: *P* < 0.001. Data are presented as mean ± SEM.

Abbreviations: BS, treatment-naïve baseline; nCRT, neoadjuvant chemoradiotherapy; PPCT, preoperative pembrolizumab combined with chemoradiotherapy; vSMC, vascular smooth muscle cell.

**
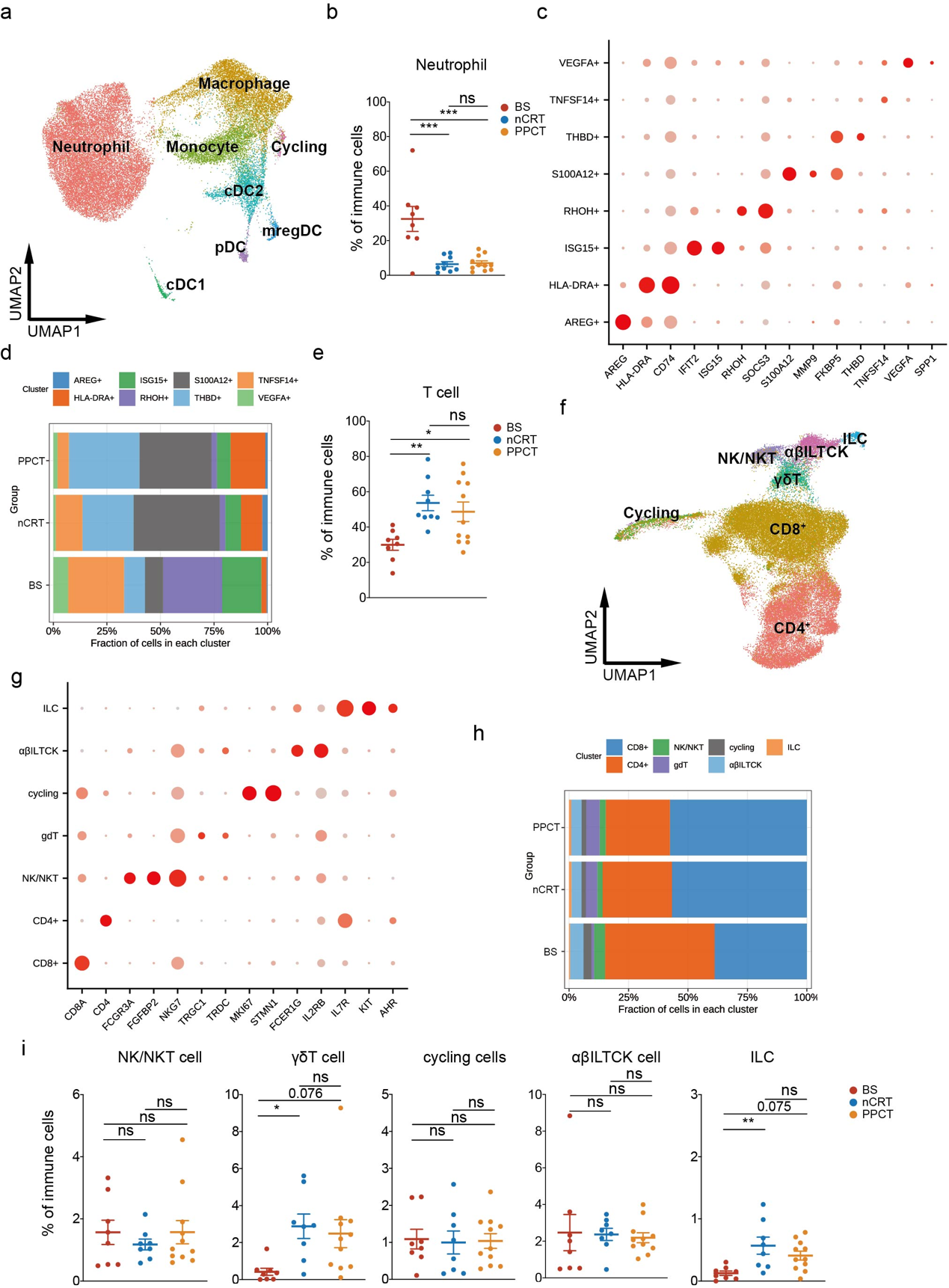
**

Supplementary Fig. 3. Neoadjuvant chemoradiotherapy alone or in combination with anti-PD-1 therapy restores the balance between T cells and neutrophils within the tumor.

(a) UMAP plots highlighting myeloid cell clusters. (b) Box plot presenting neutrophil proportions across different groups. (c) Dotplot illustrating the expression levels of key marker genes that define each neutrophil cluster. (d) Stacked bar chart comparing the neutrophil proportions among different groups. (e) Box plot illustrating T-cell infiltration across different groups. (f) UMAP plots visualizing T cell clusters. (g) Dotplot illustrating the expression levels of key marker genes that define each T-cell cluster. (h) Stacked bar chart and (i) box plot comparing the T-cell proportions among different groups. *P* values were derived from one-way ANOVA, Tukey’s test; ns: not significant, *: *P*<0·05, **: *P*<0·01, ***: *P*<0·001. Data are presented as mean ± SEM.

Abbreviations: BS, treatment-naïve baseline; nCRT, neoadjuvant chemoradiotherapy; PPCT, preoperative pembrolizumab combined with chemoradiotherapy.


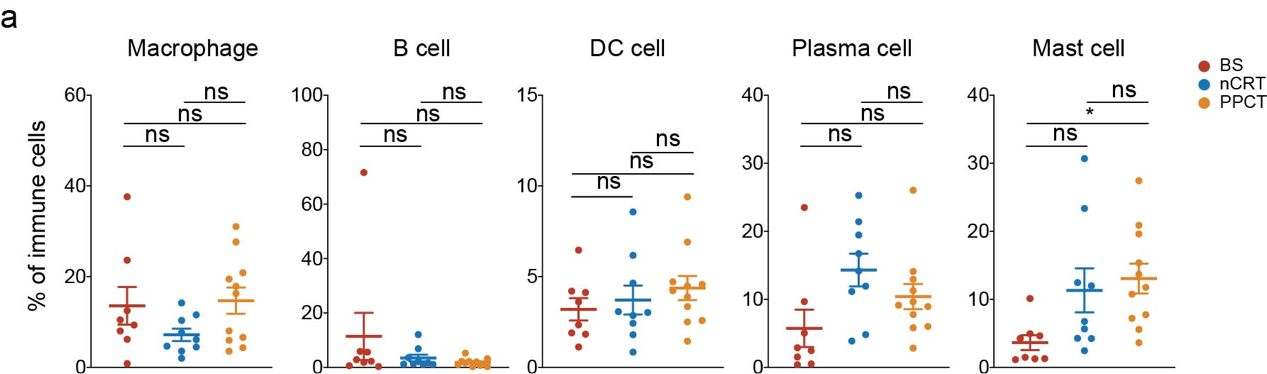


Supplementary Fig. 4. PPCT has no effect on other immune cell subsets.

(a) Box plots showing the proportions of other immune cell types across different groups. *P* values were derived from one-way ANOVA, Tukey’s test; ns: not significant, *: *P*<0·05. Data are presented as mean ± SEM.

Abbreviations: BS, treatment-naïve baseline; nCRT, neoadjuvant chemoradiotherapy; PPCT, preoperative pembrolizumab combined with chemoradiotherapy.

**
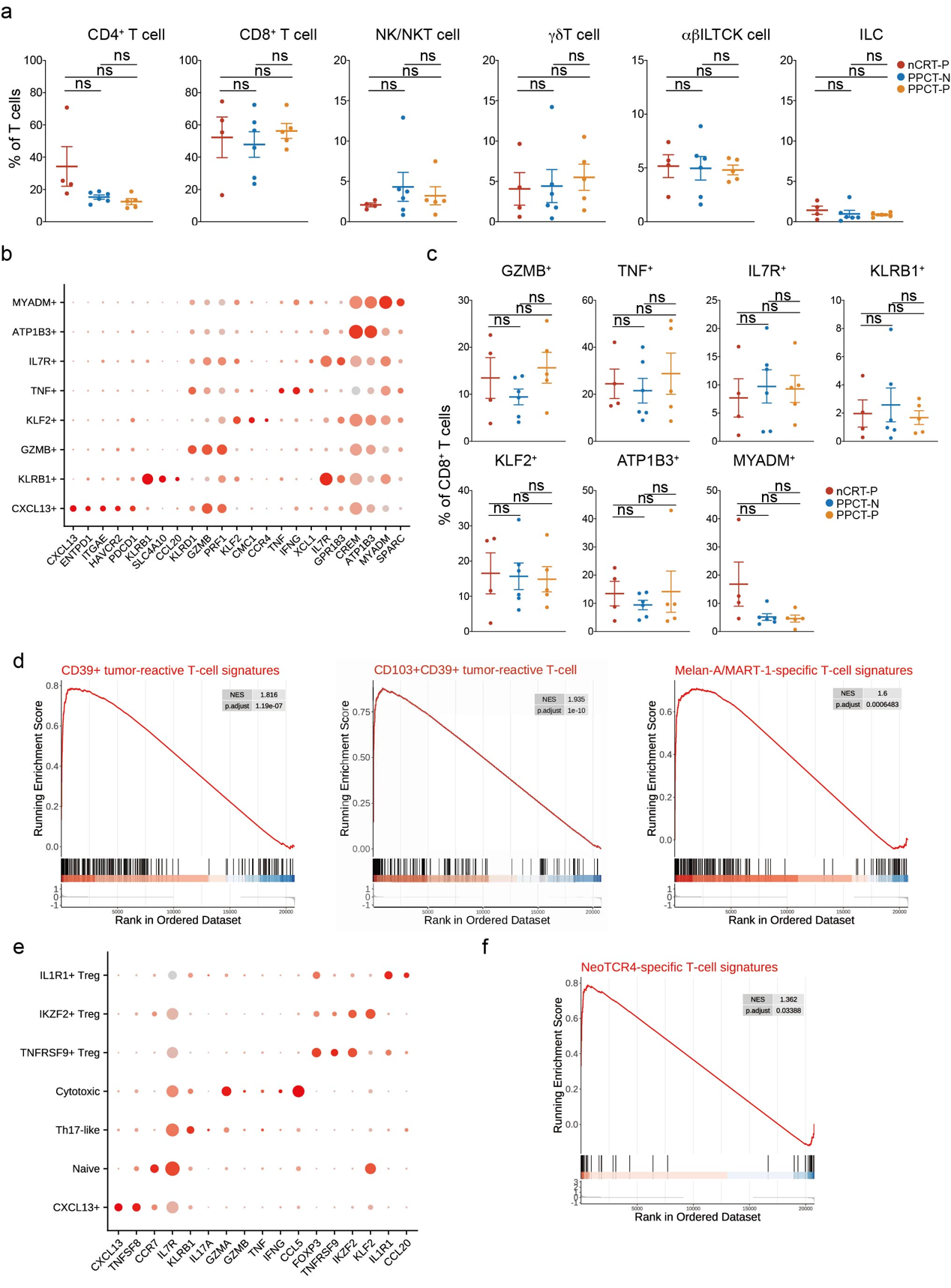
**

Supplementary Fig. 5. Supplementing anti-PD-1 blockade induces a robust anti-tumor T cell response.

(a) Box plots showing the proportions of T-cell types across different groups. (b) Dotplot displaying the expression of specific genes of CD8^+^ T-cell clusters. (c) Box plots showing the proportions of CD8^+^ T-cell clusters across different groups. (d) GSEA of the CXCL13^+^CD8^+^ subset for three signatures of tumor-reactive CD8^+^ T cells. (e) Dotplot displaying the expression of specific genes of CD4^+^ T-cell clusters. (f) GSEA of the CXCL13^+^CD4^+^ subset for signature of tumor-reactive CD4^+^ T cells. *P* values were derived from one-way ANOVA, Tukey’s test; ns: not significant. Data are presented as mean ± SEM. *P* values for GSEA were determined by permutation test according to the standard GSEA procedure.

Abbreviations: BS, treatment-naïve baseline; nCRT, neoadjuvant chemoradiotherapy; PPCT, preoperative pembrolizumab combined with chemoradiotherapy; PPCT-N, non-pCR following PPCT; PPCT-P, pCR following PPCT; pCR, pathological complete response; GSEA, gene set enrichment analysis.

**
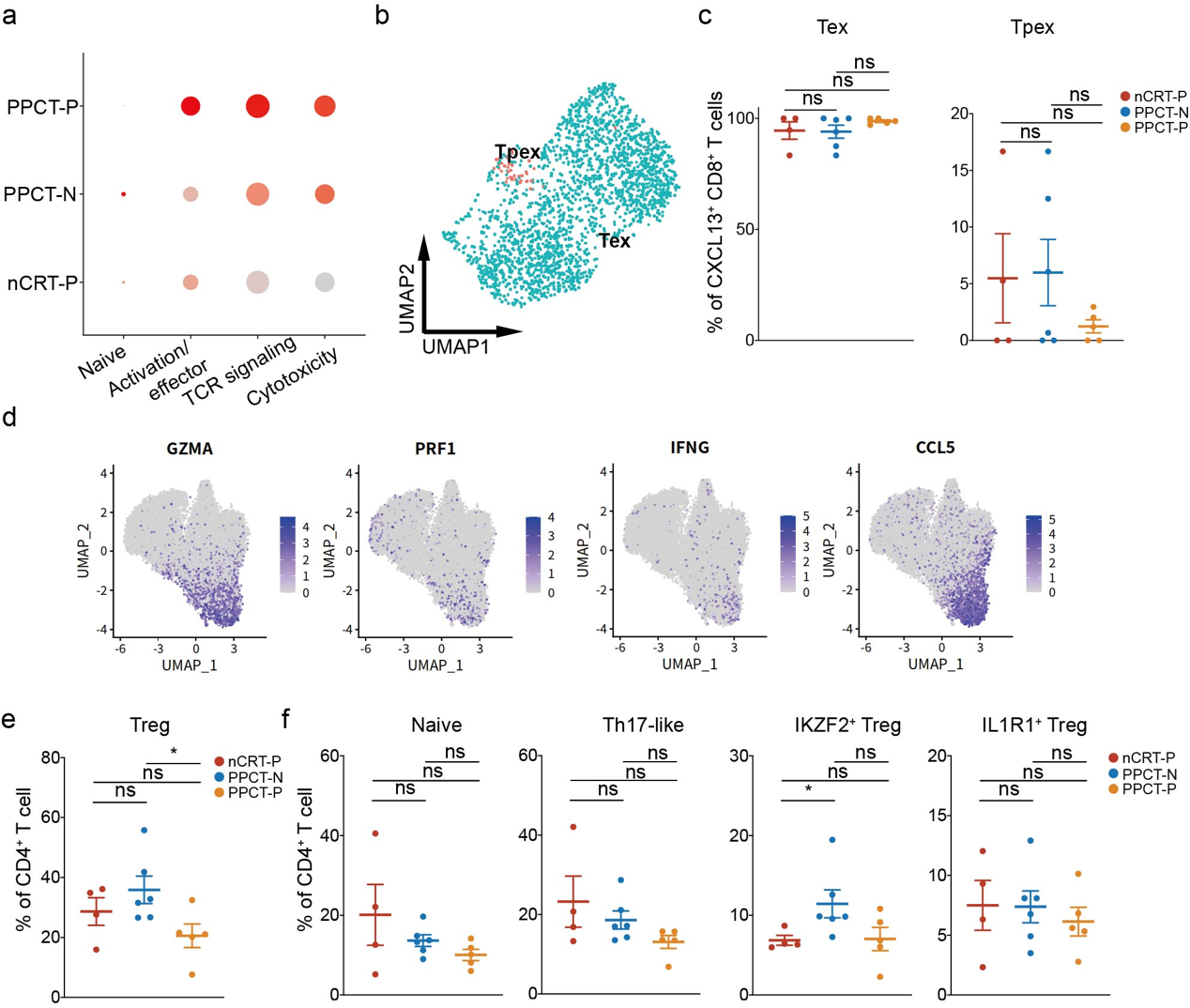
**

Supplementary Fig. 6. Additional changes of T cell clusters in pCR samples after PPCT.

(a) Dotplot comparing signature scores of CD8^+^ functions across different groups. (b) UMAP plots illustrating the CXCL13^+^CD8^+^ clusters. (c) Box plots comparing the proportions of CXCL13^+^CD8^+^ clusters across different groups. (d) UMAP plots illustrating the marker genes of cytotoxic CD4^+^ T cells. (e, f) Box plots showing the proportions of (e) total Treg cell and (f) CD4^+^ T-cell types across different groups. *P* values were derived from one-way ANOVA, Tukey’s test; ns: not significant, *: *P*<0·05. Data are presented as mean ± SEM.

Abbreviations: BS, treatment-naïve baseline; nCRT, neoadjuvant chemoradiotherapy; PPCT, preoperative pembrolizumab combined with chemoradiotherapy; PPCT-N, non-pCR following PPCT; PPCT-P, pCR following PPCT; pCR, pathological complete response; Tex, terminally exhausted CD8^+^ T cells; Tpex, precursor exhausted CD8^+^ T cells.

**
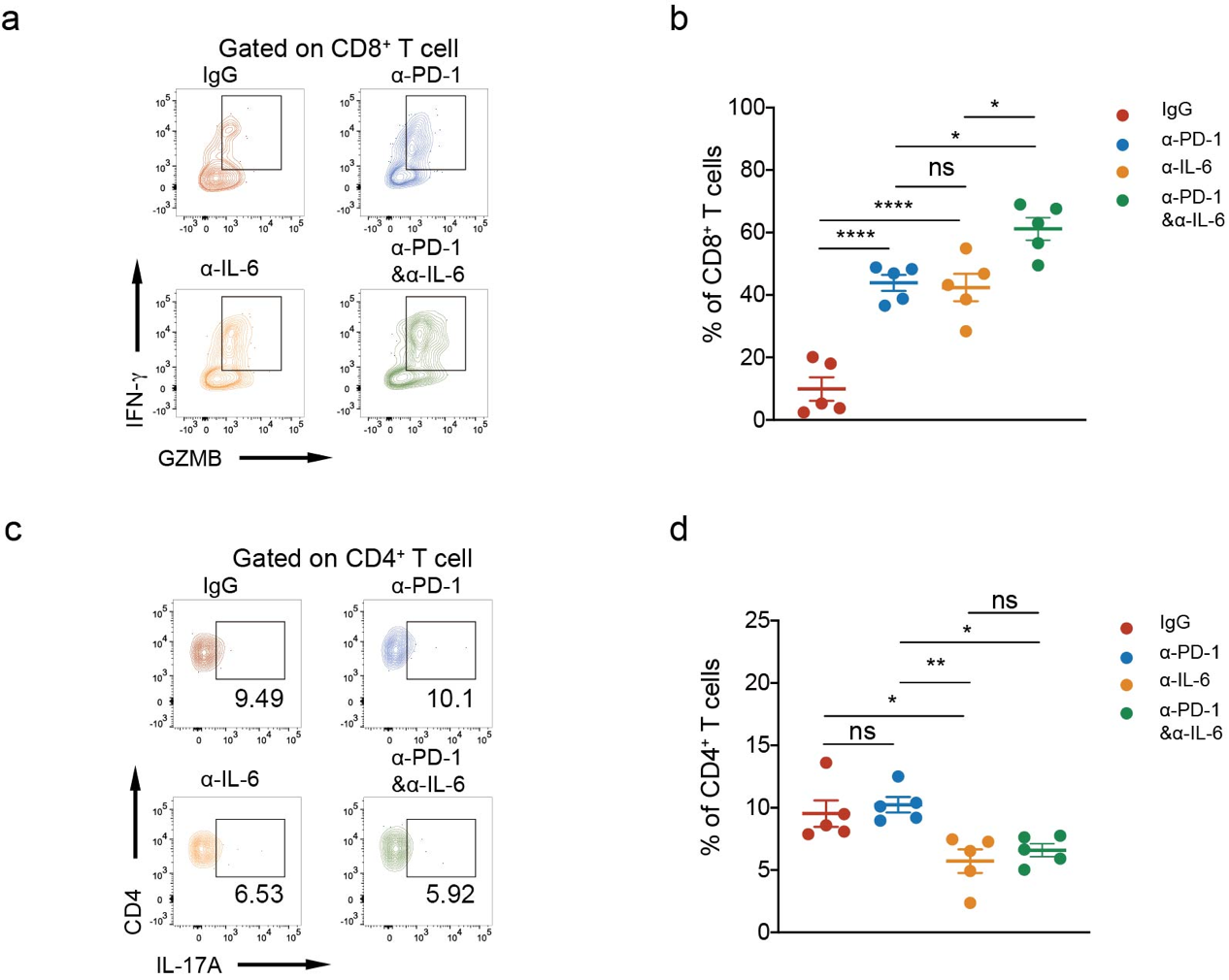
**

Supplementary Fig. 7. T-cell alterations in the murine tumor model treated with anti-PD-1 and IL-6 blockade.

(a) Proportion of IFNγ- and GZMB-expressing CD8^+^ T cells in the tumor by flow cytometry. (b) Statistical summary of IFN-γ^+^GZMB^+^CD8^+^ T cells analyzed by flow cytometry. (c) Proportion of Th17 cells by flow cytometry. (d) Statistical summary of alterations in Th17 cells analyzed by flow cytometry. *P* values were derived from one-way ANOVA, Tukey’s test; ns: not significant, *: *P*<0·05, **: *P*<0·01, ****: *P*<0·0001. Data are presented as mean ± SEM.

Supplementary Table 1.

Baseline Demographics and Clinical Characteristics (n = 140).

| **Characteristics** | **Overall**  **n = 140, (%)** | **pCR**  **n = 54, (%)** | **non-pCR**  **n = 71, (%)** | ***P* value** |
| --- | --- | --- | --- | --- |
| **Age, year** |  |  |  | 0.15 |
| Median | 63 | 63 | 64 |  |
| IQR | 11 | 9 | 9.5 |  |
| **Sex** |  |  |  | 0.81 |
| Male | 117 (83.6) | 44 (81.5) | 60 (84.5) |  |
| Female | 23 (16.4) | 10 (18.5) | 11 (15.5) |  |
| **ECOG performance status** |  |  |  | 0.02 |
| 0 | 17 (12.1) | 12 (22.2) | 5 (7.0) |  |
| 1 | 123 (87.9) | 42 (77.8) | 66 (93.0) |  |
| **BMI, kg/m^2^** |  |  |  | 0.54 |
| Mean | 22.97 | 23.18 | 22.87 |  |
| SD | 2.91 | 2.90 | 2.79 |  |
| **History of smoking** |  |  |  | 1.00 |
| Yes | 99 (70.7) | 33 (61.1) | 49 (69.0) |  |
| No | 41 (29.3) | 21 (38.9) | 22 (31.0) |  |
| **Tumor length, cm** |  |  |  | 0.69 |
| Median | 2.68 | 2.75 | 2.43 |  |
| IQR | 1.67 | 1.81 | 1.54 |  |
| **Tumor location** |  |  |  | 0.49 |
| Proximal third | 8 (5.7) | 5 (9.26) | 3 (42.3) |  |
| Middle third | 51 (36.4) | 19 (35.2) | 24 (33.8) |  |
| Distal third | 78 (55.7) | 28 (51.9) | 43 (60.6) |  |
| Cardia | 3 (2.1) | 2 (3.7) | 1 (1.4) |  |
| **Clinical T stage** |  |  |  | 0.68 |
| cT2 | 14 (10.0) | 7 (13.0) | 6 (8.5) |  |
| cT3 | 97 (69.3) | 35 (64.8) | 50 (70.4) |  |
| cT4a | 29 (20.7) | 12 (22.2) | 15 (21.1) |  |
| **Clinical N stage** |  |  |  | 0.03 |
| cN0 | 32 (22.9) | 7 (13.0) | 21 (29.6) |  |
| cN1 | 64 (45.7) | 26 (48.1) | 31 (43.7) |  |
| cN2 | 39 (27.9) | 21 (38.9) | 16 (22.5) |  |
| cN3 | 5 (3.6) | 0 | 3 (4.2) |  |
| **Clinical stage** |  |  |  | 0.74 |
| II | 36 (25.7) | 12 (22.2) | 20 (28.2) |  |
| III | 73 (52.1) | 30 (55.6) | 35 (49.3) |  |
| IVA | 31 (22.1) | 12 (22.2) | 16 (22.5) |  |

Supplementary Table 2.

Recurrence Patterns in 22 Patients.

| **Recurrence** | **n (%)** |
| --- | --- |
| **Locoregional recurrence** |  |
| Mediastinal | 9 (40.9) |
| Abdominal | 1 (4.5) |
| Multiple | 0 (0.0) |
| **Distant metastasis** |  |
| Lung | 4 (18.2) |
| Liver | 1 (4.5) |
| Bone | 1 (4.5) |
| Lung + liver | 1 (4.5) |
| **Combined recurrence** |  |
| Liver + regional lymph node | 3 (13.6) |
| Bone + regional lymph node | 1 (4.5) |
| Lung + regional lymph node + pleura | 1 (4.5) |

Supplemental Table 3.

Clinical characteristics of 28 patients with esophageal squamous cell carcinoma for Analysis.

| **Sample ID** | **Group** | **Subgroup** | **Regimen** | **TRG** | **pCR/ non-pCR** | **Location** | **Histology** | **TNM stage** |
| --- | --- | --- | --- | --- | --- | --- | --- | --- |
| BS-01 | BS | BS | NA | NA | / | Middle | ESCC | pT2N0M0 |
| BS-02 | BS | BS | NA | NA | / | Distal | ESCC | pT1bN0M0 |
| BS-03 | BS | BS | NA | NA | / | Middle | ESCC | pT3N0M0 |
| BS-04 | BS | BS | NA | NA | / | Distal | ESCC | pT3N2M0 |
| BS-05 | BS | BS | NA | NA | / | Middle | ESCC | pT2N0M0 |
| BS-06 | BS | BS | NA | NA | / | Distal | ESCC | pT1bN0M0 |
| BS-07 | BS | BS | NA | NA | / | Distal | ESCC | pT2N0M0 |
| BS-08 | BS | BS | NA | NA | / | Proximal | ESCC | pT2N1M0 |
| nCRT-01 | CR | nCRT-N | CROSS | 1 | non-pCR | Middle | ESCC | ypT2N0M0 |
| nCRT-02 | CR | nCRT-N | CROSS | 1 | non-pCR | Distal | ESCC | ypT2N0M0 |
| nCRT-03 | CR | nCRT-N | CROSS | 2 | non-pCR | Proximal | ESCC | ypT3N0M0 |
| nCRT-04 | CR | nCRT-N | CROSS | 2 | non-pCR | Proximal | ESCC | ypT3N0M0 |
| nCRT-05 | CR | nCRT-N | CROSS | 1 | non-pCR | Distal | ESCC | ypT1bN0M0 |
| nCRT-06 | CR | nCRT-P | CROSS | 0 | pCR | Distal | ESCC | ypTxN0M0 |
| nCRT-07 | CR | nCRT-P | CROSS | 0 | pCR | Distal | ESCC | ypT0N0M0 |
| nCRT-08 | CR | nCRT-P | CROSS | 0 | pCR | Distal | ESCC | ypT0N0M0 |
| nCRT-09 | CR | nCRT-P | CROSS | 0 | pCR | Middle | ESCC | ypT0N0M0 |
| PPCT-01 | CRI | PPCT-N | PALACE | 1 | non-pCR | Middle | ESCC | ypT1aN0M0 |
| PPCT-02 | CRI | PPCT-N | PALACE | 1 | non-pCR | Distal | ESCC | ypT2N1M0 |
| PPCT-03 | CRI | PPCT-N | PALACE | 1 | non-pCR | Middle | ESCC | ypT2N0M0 |
| PPCT-04 | CRI | PPCT-N | PALACE | 2 | non-pCR | Middle | ESCC | ypT3N0M0 |
| PPCT-05 | CRI | PPCT-N | PALACE | 1 | non-pCR | Middle | ESCC | ypT1bN1M0 |
| PPCT-06 | CRI | PPCT-N | PALACE | 2 | non-pCR | Distal | ESCC | ypT3N2M0 |
| PPCT-07 | CRI | PPCT-P | PALACE | 0 | pCR | Middle | ESCC | ypT0N0M0 |
| PPCT-08 | CRI | PPCT-P | PALACE | 0 | pCR | Distal | ESCC | ypT0N0M0 |
| PPCT-09 | CRI | PPCT-P | PALACE | 0 | pCR | Distal | ESCC | ypT0N0M0 |
| PPCT-10 | CRI | PPCT-P | PALACE | 0 | pCR | Middle | ESCC | ypT0N0M0 |
| PPCT-11 | CRI | PPCT-P | PALACE | 0 | pCR | Distal | ESCC | ypT0N0M0 |

*BS = treatment-naïve baseline; *nCRT = chemoradiotherapy; *PPCT = preoperative pembrolizumab combined with chemoradiotherapy

* nCRT-N = non-pCR after nCRT; * nCRT-P = pCR after nCRT; * PPCT-N = non-pCR after PPCT; * PPCT-P = pCR after PPCT

*ESCC = esophageal squamous cell carcinoma

*TRG grade = Tumor regression grade

*CROSS regimen of chemoradiotherapy refers to that in the paper of CROSS trial

Supplemental Table 4.

Comparison of clinical characteristics of patients between different groups.

| **Parameters** | **BS vs nCRT vs PPCT** | | | | **PPCT-N vs PPCT-P** | | |
| --- | --- | --- | --- | --- | --- | --- | --- |
|  | **BS (n=8)** | **nCRT (n=9)** | **PPCT (n=11)** | ***P* value** | **PPCT-N (n=6)** | **PPCT-P (n=5)** | ***P* value** |
| **Sex, n (%)** |  |  |  | 0.38 |  |  | 0.52 |
| Male | 8 (100.0%) | 7 (77.8%) | 9 (81.8%) |  | 4 (66.7%) | 5 (100.0%) |  |
| Female | 0 (0.0%) | 2 (22.2%) | 2 (18.2%) |  | 2 (33.3%) | 0 (0%) |  |
| **Age (yr), mean ± SD** | 64.6 ± 10.2 | 64.9 ± 5.0 | 61.4 ± 6.4 | 0.49 | 62.3 ± 6.1 | 60.2 ± 7.3 | 0.62 |
| **Location, n (%)** |  |  |  | 0.44 |  |  | 0.78 |
| Distal third | 4 (50.0%) | 5 (55.6%) | 5 (45.5%) |  | 2 (33.3%) | 3 (60.0%) |  |
| Middle third | 3 (37.5%) | 2 (22.2%) | 6 (54.5%) |  | 4 (66.7%) | 2 (40.0%) |  |
| Proximal third | 1 (12.5%) | 2 (22.2%) | 0 (0%) |  | 0 (0%) | 0 (0%) |  |

*SD = standard deviation

*****BS = treatment-naïve baseline; *nCRT = chemoradiotherapy; *PPCT = preoperative pembrolizumab combined with chemoradiotherapy

* nCRT-N = non-pCR after nCRT; * nCRT-P = pCR after nCRT; * PPCT-N = non-pCR after PPCT; * PPCT-P = pCR after PPCT

*Pearson’s Chi-squared test or Fisher’s exact test was applied for comparing categorical variables.

*Two-sided unpaired Student’s t test and one-way analysis of variance test were used for comparing continous variables.

1. van Hagen, P. *et al.* Preoperative chemoradiotherapy for esophageal or junctional cancer. *N Engl J Med* **366**, 2074-2084 (2012).

2. Kojima, T. *et al.* Randomized Phase III KEYNOTE-181 Study of Pembrolizumab Versus Chemotherapy in Advanced Esophageal Cancer. *J Clin Oncol*, Jco2001888 (2020).

3. Ajani, J.A. *et al.* Esophageal and Esophagogastric Junction Cancers, Version 2.2023, NCCN Clinical Practice Guidelines in Oncology. *J Natl Compr Canc Netw* **21**, 393-422 (2023).

4. Yang, H. *et al.* Neoadjuvant Chemoradiotherapy Followed by Surgery Versus Surgery Alone for Locally Advanced Squamous Cell Carcinoma of the Esophagus (NEOCRTEC5010): A Phase III Multicenter, Randomized, Open-Label Clinical Trial. *J Clin Oncol* **36**, 2796-2803 (2018).

5. Li, C. *et al.* Preoperative pembrolizumab combined with chemoradiotherapy for oesophageal squamous cell carcinoma (PALACE-1). *Eur J Cancer* **144**, 232-241 (2021).
