# Supplementary material for: Preoperative pembrolizumab (anti-PD-1 antibody) combined with chemoradiotherapy for esophageal squamous cell carcinoma: a phase 1/2 trial (PALACE-2)
Source: Signal Transduct Target Ther. 2025 Nov 28;10:386. doi: 10.1038/s41392-025-02477-4 (PMC12660869; doi:10.1038/s41392-025-02477-4)
Supplement: Supplementary file 2 — Supplementary information [file 41392_2025_2477_MOESM2_ESM.docx]

**Study Protocol**

**Preoperative pembrolizumab combined with chemoradiotherapy for esophageal squamous cell carcinoma.**

**A muti-center phase 1/2 study**

**Version 1.1, DATE: August 2020**

| Study Number： | NCT03792347 (phase 1)  NCT04435197 (phase 2) |
| --- | --- |
| Principal Investigator： | Hecheng Li |
| Contact Information： | Tel: (+86) 021-64370045  Email: [lihecheng2000@hotmail.com](mailto:lihecheng2000@hotmail.com) |
| Address： | Department of Thoracic Surgery, Ruijin Hospital, Shanghai Jiao Tong University School of Medicine, 197 Ruijin 2nd Road, Shanghai 200025, China |

| **CONFIDENTIAL**  This document is confidential, and the information contained therein belongs to the Sponsor. It is prohibited to view or disclose any relevant information without the written approval of the sponsor. The information is for use only by those who approved or conducted this study.  **STATEMENT**  This study was conducted in strict accordance with ICH GCP E6 (R2), Good Clinical Practice (GCP), and appropriate regulatory requirements. All requested research documents will be filed as required. |
| --- |

# Signature page

（Investigator）

I have read and understand the protocol and agree that it contains the ethical, legal, and scientific information necessary to participate in this study. My signature confirms my agreement that the study will be conducted in accordance with the protocol and all applicable laws and regulations including, but not limited to, Good Pharmacoepidemiology Practices (GPP), the ethical principles that have their origins in the Declaration of Helsinki, and applicable privacy laws.

I will provide copies of this protocol as needed to all physicians, nurses, and other professional personnel responsible to me who will participate in the Study. I will discuss the protocol with them to assure myself that they are sufficiently informed regarding the conduct of the Study. I am aware that this protocol will need to be approved by an appropriate Independent Ethics Committee (IEC) prior to any subjects being enrolled and that I am responsible for verifying whether that requirement is met. I agree to adhere to the attached protocol and if requested to provide copies of medical information for the purpose of verification of submitted information, I will comply.

Since the information in this protocol is confidential, I understand that its disclosure to any third parties, other than those involved in approval, supervision, or conduct of the study is prohibited. I will ensure that the necessary precautions are taken to protect such information from loss, inadvertent disclosure, or access by third parties.

Principal Investigator：Hecheng Li

Study site (group leader)：Hecheng Li

| Signature： | Date：2020-08-30 |
| --- | --- |

# Synopsis

| **Study title** | Preoperative pembrolizumab combined with chemoradiotherapy for esophageal squamous cell carcinoma. |
| --- | --- |
| **Study type** | Prospective, multi-center, single-arm, phase 1/2 study |
| **Objectives** | The primary study parameter of PALACE-2 trial is the pCR rate.  pCR is defined as the absence of any signs of cancer in resected tissue samples examined by pathologists.  Secondary  The secondary study parameters include:  _ Three-year DFS rate;  _ Three-year OS rate;  _ R0 resection rate, defined as the percentage of patients who undergo surgery and achieve a tumor-free resection margin;  _ Rate of AEs during neoadjuvant therapy and perioperative period.  AEs will be evaluated and recorded according to the NCICTCAE (version 5.0). |
| **Study design** | The PALACE-2 study is a prospective, multicenter, single-arm clinical trial, with an estimated inclusion period of 3 years, the primary end point is anticipated to be achieved in June 2023. |
| **Rationale** | Immunotherapy is a promising treatment for advanced ESCC. Meanwhile, preclinical studies have also proven the synergy between ICI and chemoradiotherapy.  Therefore, it is reasonable to evaluate the addition of immunotherapy to the neoadjuvant regimen for locally advanced disease. There are several ongoing prospective clinical trials that focus mainly on the combination of immunotherapy and neoadjuvant chemotherapy/nCRT for esophageal cancer. In the recently completed Preoperative Anti-PD-1 Antibody combined with Chemoradiotherapy for Locally Advanced Squamous Cell Carcinoma of Esophagus (PALACE)-1 trial, we investigated the safety and feasibility of preoperative pembrolizumab combined with chemoradiotherapy (PPCT) followed by surgery in treating locally advanced ESCC. Twenty patients were enrolled, and 18 underwent surgery. PPCT was shown to be safe and feasible and induced a pCR in 55.6% of resected specimens.  The results of PALACE-1 study justify a further clinical trial. Therefore, we design and conduct a subsequent multicenter single-arm PALACE-2 trial to investigate the efficacy and to further confirm the safety of PPCT. |
| **Therapy**  **schedule** | Administration of Paclitaxel / Nab-paclitaxel and Carboplatin  Paclitaxel (phase 1) or Nab-paclitaxel (phase 2) 50 mg/m^2^ and Carboplatin AUC = 2 will be given by intravenous infusion on days 1, 8,15, 22 and 29.  Radiotherapy schedule  A total dose of 41.4 Gy will be given in 23 fractions of 1.8 Gy, 5 fractions per week, starting the first day of the first cycle of chemotherapy. All patients will be radiated by external beam radiation, using 3-D conformal radiation technique.  Administration of Pembrolizumab  Pembrolizumab will be given concurrently on day 1 and 22 of the neoadjuvant therapy at a dose of 200 mg. For patients weighing < 50 kg, pembrolizumab will be given at a dose of 100 mg.  Surgery  Surgery should be arranged 4 to 6 weeks after completion of PPCT. |
| **Inclusion**  **criteria** | _ Histologically confirmed ESCC, with a clinical stage of cT2 though T4a, N0 through N3, M0;  _ Age ranging from 18 to 75 years;  _ Eastern Cooperative Oncology Group performance status score of 0 to 1;  _ Patients approve and sign the informed consent. |
| **Exclusion**  **criteria** | _ Patients with active autoimmune diseases or history of autoimmune diseases;  _ Patients who need systemic treatment with either corticosteroids or other immunosuppressive drugs;  _ Patients with symptomatic interstitial pulmonary disease;  _ Patients who are allergic to drugs used in the trial;  _ Pregnant or lactating women;  _ Patients of childbearing age who are not willing to use contraceptive measures;  _Patients who have previously received targeted therapy, immunotherapy, chemotherapy, or radiotherapy for this or any other prior malignancies;  _ Underlying medical conditions that, in the investigator’s opinion, will increase the risk of medication use or obscure the interpretation of toxicity and AEs. |
| **Sample size** | According to a review of current literature, the pCR rate after neoadjuvant chemoradiotherapy for locally advanced ESCC in Asian population was expected to be 43.2%. Meanwhile, the pCR rate after PPCT was assumed to be 56%, based on the short-term results of our prior PALACE-1 trial. With a power of 80%, a sample size of 130 will be required to detect a difference in pCR rate between PPCT and chemoradiotherapy at a significance level of 5%. To allow for a 10% of dropout cases, the sample size was increased to 143 in this PALACE-2 trial. |
| **Duration of the**  **study (planned)** | This study was initiated during January 2019. With an estimated inclusion period of about 4 years, the primary end point is anticipated to be achieved in June 2023. |

Table of contents

[Signature page 3](#_Toc178496103)

[Synopsis 4](#_Toc178496104)

[Table of contents 7](#_Toc178496105)

[List of figures 10](#_Toc178496106)

[List of tables 10](#_Toc178496107)

[Relevant Abbreviations 11](#_Toc178496108)

[1 Introduction 13](#_Toc178496109)

[2 Objectives of the study 14](#_Toc178496110)

[3 Design of the study 15](#_Toc178496111)

[3.1 Overall design 15](#_Toc178496112)

[3.2 Duration of the study 16](#_Toc178496113)

[3.3 Number of patients and recruitment 16](#_Toc178496114)

[4 Patient selection criteria 16](#_Toc178496115)

[4.1 Inclusion criteria 16](#_Toc178496116)

[4.2 Exclusion criteria 16](#_Toc178496117)

[4.3 Exit criteria 17](#_Toc178496118)

[4.3.1 Screening failure 17](#_Toc178496119)

[4.3.2 Withdrawal of informed consent 17](#_Toc178496120)

[4.3.3 Discontinue the study 17](#_Toc178496121)

[5 Treatment 17](#_Toc178496122)

[5.1 Chemotherapy regimen 18](#_Toc178496123)

[5.2 Radiotherapy schedule 19](#_Toc178496124)

[5.3 Immunotherapy regimen 21](#_Toc178496125)

[5.4 Surgery 21](#_Toc178496126)

[5.5 Dose modifications / Retreatment delay 22](#_Toc178496127)

[5.5.1 Chemotherapeutic toxicity 22](#_Toc178496128)

[5.5.2 Radiation toxicity 24](#_Toc178496129)

[5.5.3 Immune-related toxicity 25](#_Toc178496130)

[5.6 Labeling 25](#_Toc178496131)

[5.7 Storage 25](#_Toc178496132)

[5.8 Drug inventory 25](#_Toc178496133)

[5.9 Compliance 25](#_Toc178496134)

[6 Study procedures 26](#_Toc178496135)

[6.1 Treatment plan 26](#_Toc178496136)

[6.2 Baseline evaluation 27](#_Toc178496137)

[6.3 Follow-up 27](#_Toc178496138)

[6.4 Patient discontinuation 27](#_Toc178496139)

[6.5 Effectiveness evaluation 28](#_Toc178496140)

[6.6 Security evaluation 29](#_Toc178496141)

[7 Concomitant therapy 29](#_Toc178496142)

[8 Data management 30](#_Toc178496143)

[8.1 Data collection 30](#_Toc178496144)

[8.2 Confidentiality 30](#_Toc178496145)

[8.3 Investigator site file 30](#_Toc178496146)

[9 Statistical analysis 30](#_Toc178496147)

[10 Assessment of response and toxicity 31](#_Toc178496148)

[11 Ethical consideration 31](#_Toc178496149)

[12 Safey 32](#_Toc178496150)

[12.1 Definitions 32](#_Toc178496151)

[12.1.1 Adverse event 32](#_Toc178496152)

[12.1.2 Serious adverse event 32](#_Toc178496153)

[12.2 Collection of adverse events 32](#_Toc178496154)

[12.2.1 Collection period 32](#_Toc178496155)

[12.2.2 Collection method 33](#_Toc178496156)

[12.3 Follow-up of Adverse Events 33](#_Toc178496157)

[12.4 Record and assessment of adverse event 33](#_Toc178496158)

[12.4.1 Record 33](#_Toc178496159)

[12.4.2 Assessment of severity 34](#_Toc178496160)

[12.4.3 Assessment of causality 34](#_Toc178496161)

[12.4.4 Outcome 36](#_Toc178496162)

[12.4.5 Disease progression 37](#_Toc178496163)

[12.4.6 New tumors 37](#_Toc178496164)

[12.4.7 Death 37](#_Toc178496165)

[12.4.8 AESI 37](#_Toc178496166)

[12.5 Reports of adverse event 38](#_Toc178496167)

[12.5.1 Serious adverse event reporting requirements 38](#_Toc178496168)

[12.5.2 Drug overdose 38](#_Toc178496169)

[12.6 Pregnancy 38](#_Toc178496170)

[13 Entry and registration procedures 39](#_Toc178496171)

[14 Address list 39](#_Toc178496172)

[15 Administrative and legal obligations 39](#_Toc178496173)

[15.1 Protocol amendments 39](#_Toc178496174)

[15.2 Trial documentation and data storage 40](#_Toc178496175)

[15.3 Trial termination 40](#_Toc178496176)

[16 Publication and registration of the study 40](#_Toc178496177)

[17 References 42](#_Toc178496178)

# List of figures

# 未找到图形项目表。

[Figure 1: Trial design and flow chart 15](#_Toc212028975)

# List of tables

[Table 1: Treatment Schedule 26](#_Toc212028976)

# Relevant Abbreviations

| AE | Adverse Event | nCRT | neoadjuvant chemoradiotherapy |
| --- | --- | --- | --- |
| AESI | Adverse Event Special Interest | NCICTCAE | National Cancer Institute-Common Terminology Criteria for Adverse Events |
| CROSS | Chemoradiotherapy for Oesophageal  Cancer Followed by Surgery Study | NMPA | National Medical Products Administration |
| CRC | Clinical Coordinator | OS | Overall Survival |
| CRF | Case Report Form | PALACE | Preoperative Anti-PD-1 Antibody combined with Chemoradiotherapy for Locally Advanced Squamous Cell Carcinoma of Esophagus |
| CRO | Contract Research Organization | pCR | Pathologic Complete Response |
| CT | Computerized Tomography | PD | Protocol Deviation |
| ESCC | Esophageal Squamous Cell Carcinoma | PD-1 | programmed cell death protein 1 |
| EC | Ethics Committee | PD-L1 | programmed cell death ligand 1 |
| ECG | Electrocardiogram | PPCT | preoperative pembrolizumab combined with chemoradiotherapy |
| ECOG | Eastern Cooperative Oncology Group | PP | Per protocol |
| eCRF | Electronic Case Report Form | PPS | Per Protocol Set |
| EDC | Electronic Data Capture | PET-CT | positron emission tomography-computed tomography |
| EOT | End of Treatment | SAE | Serious Adverse Event |
| FAS | Full Analysis Set | SAP | Statistical Analysis Plan |
| GCP | Good Clinical Practice | SAS | Statistical Analysis System |
| ICI | Immune Checkpoint Inhibitor | SD | Standard Deviation |
| ICF | Informed Consent Form | SDV | Source Document Verification |
| ICH | International Council for Harmonization | SOP | Standard Operating Procedure |
| ITT | Intention To Treat Principle | SS | Safety Analysis Set |
| MedDRA | Medical Dictionary for Regulatory Activities | TEAE | Treatment-Emergent Adverse Event |

# Introduction

Epidemiology

Esophageal cancer is the seventh most common malignancy around the world.^1^ In the Asian population, more than 90% of the diagnosed esophageal cancers are esophageal squamous cell carcinoma (ESCC).^2^ Given the difficulties of early screening, nearly half of patients are diagnosed as having locally advanced disease, and neoadjuvant chemoradiotherapy (nCRT) followed by surgery has been introduced as the recommended treatment.

Neoadjuvant chemoradiotherapy

According to the Chemoradiotherapy for esophageal Cancer Followed by Surgery Study (CROSS) trial, 29% of patients achieved pathologic complete response (pCR) after nCRT, with a substantially improved median overall survival (OS) of 48.6 months.^3, 4^

However, the 5-year OS rate was about 47%, and 49% of patients developed either local-regional recurrence or distant metastasis even after nCRT.^4^ For those patients who respond poorly to nCRT, a more effective treatment strategy is required for further improvement of survival.

nCRT combined with PD-1/PD-L1

By enhancing the antitumor activity of T cells, immunotherapy is currently considered a promising treatment for various types of malignancies, including esophageal cancer.

Given the overall high level of tumor mutation burden and the high rate of positive programmed cell death ligand 1 (PD-L1) expression,^5-9^ ESCC patients are expected to experience an inspiring clinical benefit after immunotherapy targeting programmed cell death protein 1 (PD-1)/PD-L1 checkpoints.^10-12^

A widely used immune checkpoint inhibitor (ICI) targeting PD-1, pembrolizumab has been confirmed to be effective in the treatment of advanced esophageal cancer. According to the Phase II trial KEYNOTE-180, pembrolizumab monotherapy was proven to be safe and effective for heavily pretreated ESCC, with an objective response rate of 14.3%.^13^ The further Phase III randomized controlled trial KEYNOTE-181 compared pembrolizumab with chemotherapy as second-line treatment for advanced/metastatic esophageal cancer. For patients with combined positive score (the ratio of the number of all PD-L1 expressing cells to the number of all tumor cells) ≥10 in esophageal tumor, OS was significantly prolonged after pembrolizumab treatment (9.3 vs 6.7 months; P =.0074). The response rate was also improved together with a lower frequency of adverse events (AEs).^14^

Immunotherapy combined with Chemoradiotherapy

Immunotherapy is a promising treatment for advanced ESCC. Meanwhile, preclinical studies have also proven the synergy between ICI and chemoradiotherapy.^15, 16^

Therefore, it is reasonable to evaluate the addition of immunotherapy to the neoadjuvant regimen for locally advanced disease. There are several ongoing prospective clinical trials that focus mainly on the combination of immunotherapy and neoadjuvant chemotherapy/nCRT for esophageal cancer. In the recently completed Preoperative Anti-PD-1 Antibody combined with Chemoradiotherapy for Locally Advanced Squamous Cell Carcinoma of Esophagus (PALACE)-1 trial, we investigated the safety and feasibility of preoperative pembrolizumab combined with chemoradiotherapy (PPCT) followed by surgery in treating locally advanced ESCC. Twenty patients were enrolled, and 18 underwent surgery. PPCT was shown to be safe and feasible and induced a pCR in 55.6% of resected specimens.^17^

The results of PALACE-1 study justify a further clinical trial. Therefore, we designed and conducted a subsequent multicenter single-arm PALACE-2 trial to investigate the efficacy and to further confirm the safety of PPCT.

# Objectives of the study

The primary study parameter of PALACE-2 trial is the pCR rate.

pCR is defined as the absence of any signs of cancer in resected tissue samples examined by pathologists.

Secondary
The secondary study parameters include:

_ Three-year DFS rate;

_ Three-year OS rate;

_ R0 resection rate, defined as the percentage of patients who undergo surgery and achieve a tumor-free resection margin;

_ Rate of AEs during neoadjuvant therapy and perioperative period. AEs will be evaluated and recorded according to the National Cancer Institute Common Terminology Criteria for Adverse Events (version 5.0).

# Design of the study

## Overall design

The PALACE-2 study is a prospective, multicenter, single-arm clinical trial. Three medical centers in China are participating in this study (Ruijin Hospital, Shanghai Jiao Tong University School of Medicine; Cancer Hospital, Chinese Academy of Medical Sciences; and the First Affiliated Hospital of Nanchang University). This study was initiated during January 2019. With an estimated inclusion period of about 4 years, the primary end point is anticipated to be achieved in June 2023.


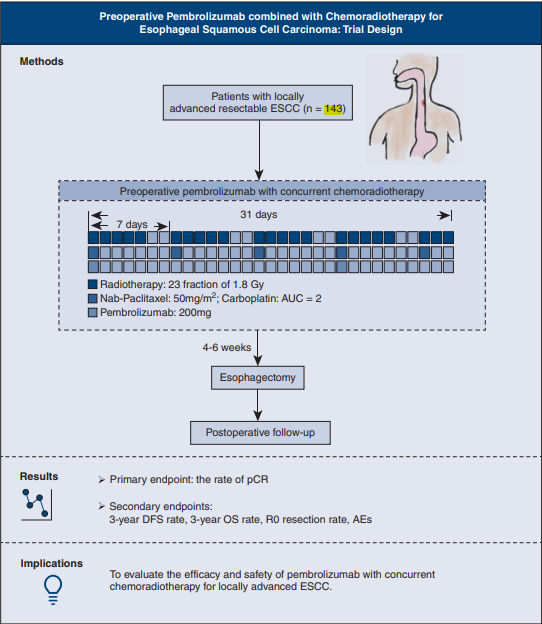


Figure 1: Trial design and flow chart

## Duration of the study

This study was initiated during January 2019. With an estimated inclusion period of about 4 years, the primary end point is anticipated to be achieved in June 2023.

## Number of patients and recruitment

The participating hospitals are estimated to recruit a total of 143 patients.

# Patient selection criteria

Patients with histologically conformed, locally advanced, and surgically resectable ESCC will be enrolled in this study. Positive PD-L1 expression is not mandatory for enrollment.

## Inclusion criteria

_ Histologically confirmed ESCC, with a clinical stage of cT2 though T4a, N0 through N3, M0;

_ Age ranging from 18 to 75 years;

_ Eastern Cooperative Oncology Group performance status score of 0 to 1;

_ Patients approve and sign the informed consent.

## Exclusion criteria

_ Patients with active autoimmune diseases or history of autoimmune diseases;

_ Patients who need systemic treatment with either corticosteroids or other immunosuppressive drugs;

_ Patients with symptomatic interstitial pulmonary disease;

_ Patients who are allergic to drugs used in the trial;

_ Pregnant or lactating women;

_ Patients of childbearing age who are not willing to use contraceptive measures;

_ Patients who have previously received targeted therapy, immunotherapy, chemotherapy, or radiotherapy for this or any other prior malignancies;

_ Underlying medical conditions that, in the investigator’s opinion, will increase the risk of medication use or obscure the interpretation of toxicity and AEs.

## Exit criteria

### Screening failure

Screening failure is defined as patients who do not meet the eligibility criteria for study enrollment and therefore cannot receive treatment. The reason for withdrawal of these patients from the study should be recorded as "not meeting the eligibility criteria for enrollment" (i.e., the patient did not meet the required enrollment criteria/met the exclusion criteria). This reason for withdrawing from the study applies only to patients who failed the screening (i.e., those who were not enrolled).

### Withdrawal of informed consent

Patients may withdraw from the study at any time, and withdrawal from the study will not affect their further treatment.

Subjects who withdraw their informed consent are asked why they withdrew and whether any AE occurred. The AE will be followed up after the clinical study.

If a subject withdraws from the study, their screening code will not be used again. Subjects who withdraw from the study will not be replaced.

### Discontinue the study

In the sponsor's judgment, the study may be discontinued if any of the following clinically significant findings would pose undue risk to the subject:

• Meets the criteria for individual study discontinuation or is considered significant

• A causal relationship with the investigational drug was assessed

• Deemed ineligible for further study

Regardless of the reason for termination, all patient data obtained at the time of discontinuation must be recorded in the eCRF. All reasons for discontinuing treatment must be documented. When terminating a study, the sponsor will ensure that patient protection is fully considered.

# Treatment

Preoperative treatment is composed of chemotherapy, radiotherapy, and immunotherapy (Figure 1). Patients will receive PPCT, which includes concurrent pembrolizumab (200 mg on day 1 and day 22), carboplatin (area under the curve 2, once a week for 5 weeks), paclitaxel (phase 1) or nab-paclitaxel (phase 2) (50 mg/m^2^, once a week for 5 weeks), and radiotherapy (23 fractions of 1.8 Gy, 5 fractions a week). Esophagectomy will be performed within 4 to 6 weeks after the completion of PPCT.

After the completion of PPCT, physical examination, routine blood test, contrast-enhanced neck, chest, and abdominal CT, echocardiography, pulmonary function, and electrocardiogram will be undertaken to reevaluate the disease and exclude cases with any surgical contraindications.

## Chemotherapy regimen

Paclitaxel (phase 1) or Nab-paclitaxel (phase 2) 50 mg/m^2^ and Carboplatin AUC = 2 will be given by intravenous infusion on days 1, 8,15, 22 and 29.

Administration of Paclitaxel / Nab-paclitaxel and Carboplatin

Premedication:

All patients receiving Paclitaxel will receive half an hour before the start of the Paclitaxel infusion premedication according to the following schedule:

| **Paclitaxel: Premedication** | **Dosage** | **Administration Route** | **Timing (prior to Paclitaxel)** |
| --- | --- | --- | --- |
| Dexamethasone | 10 mg | IV | 0.5 hour |
| Clemastine (Tavegil) | 2 mg | IV | 0.5 hour |
| Ranitidine | 50 mg | IV | 0.5 hour |

At hour 0, the total calculated dose of Paclitaxel (phase 1 cohort) or Nab-paclitaxel (phase 2 cohort), diluted in 500 ml of normal saline will be infused over one hour.

After the completion of the Paclitaxel (phase 1 cohort) or Nab-paclitaxel (phase 2 cohort) infusion, 100 ml NaCl 0.9% will be infused over 0.5 h, followed by an infusion of 8 mg Ondansetron or its equivalent diluted in 100 ml NaCl 0.9% over 0.5 hour.

Hereafter the total calculated dose of Carboplatin, diluted in 500 ml glucose 5% will be infused over one hour (doses Carboplatin > 250 mg should be dissolved in 1000 ml glucose 5%). The absolute dose of Carboplatin will be calculated for the target AUC = 2 according to the following formula:

- the absolute dose of Carboplatin = [target AUC] x (GFR + 25).
- formula GFR = [((140 – age) x 1.23 x body weight) / serum creatinin X (0.85 (female) or 1.00 (male))]

| **Paclitaxel/Nab-paclitaxel and Carboplatin infusion scheme** | |
| --- | --- |
| - 0.5 HRS START PREMEDICATION FOR PACLITAXEL (phase 1 cohort only) | |
| 0.00 hrs | **…….mg Paclitaxel / Nab-paclitaxel**  in NaCl 0.9% 500ml |
| 1.00 hrs | NaCl 0.9% 100 ml |
| 1.5 hrs | Ondansetron (or its equivalent) 8 mg in 100 ml NaCl 0.9% |
| 2.00 hrs | **…….mg Carboplatin**  in 500 ml glucose 5% in 1 hour |

Patient monitoring

It is possible that some patients will experience asymptomatic bradycardia during the Paclitaxel/Nab-paclitaxel infusion. In addition, hypersensitivity reactions are possible and generally occur within the first few minutes of initiating the infusion. For these reasons, it is recommended that there is constant supervision and that the vital signs are monitored every fifteen minutes during Paclitaxel/Nab-paclitaxel administration. Thereafter, patients may be observed, and heart rate and blood pressure checked if necessary, according to clinical symptoms.

## Radiotherapy schedule

Fractionation schedule

A total dose of 41.4 Gy will be given in 23 fractions of 1.8 Gy, 5 fractions per week, starting the first day of the first cycle of chemotherapy. All patients will be radiated by external beam radiation, using 3-D conformal radiation technique.

Position of the patient

The patient will be positioned in supine position. Reproducibility will be assessed by orthogonal laser beams.

Definitions of target volumes and critical structures

The Gross Tumor Volume (GTV) is defined by the primary tumor and any enlarged regional lymph nodes and will be drawn on each relevant CT slice. The GTV will be determined using all available information (physical examination, endoscopy, EUS, CT-neck/chest/abdomen).

The Planning Target Volume (PTV) will provide a proximal and distal margin of 4 cm, in case of tumor extension into the stomach, a distal margin of 3 cm will be chosen. A 2 cm radial margin around the GTV will be provided to include the area of subclinical involvement around the GTV and to compensate for tumor motion and set-up variations.

Both lungs will be contoured. The heart will be contoured on all slices; its cranial border will include the infundibulum of the right ventricle and the apex of both atria, and will exclude the great vessels as much as possible. The caudal border will be defined as the lowest part of the left ventricle's inferior wall that is distinguishable from the liver. The spinal canal will be contoured and taken to represent the spinal cord.

Simulation procedure

Prior to the start of the irradiation a planning CT scan will be made from the cricoid to L1vertebra with a slice thickness of 5 mm, with the patient in treatment position. The isocenter will be determined at the planning-CT.

Radiation technique

Radiation therapy will be delivered using a multiple field technique. Treatment can be given with the combination of anterior/posterior, oblique or lateral field. Customized blocks or a multi-leaf collimator will be used to shape the treatment fields.^15^

All patients will undergo a 3D planning. Beams-eye-view (BEV) displays will be used to ensure optimal target volume coverage and optimal normal tissue sparing. The most appropriate technical solutions (e.g., beam quality, field arrangement, conformal therapy planning) will be chosen as long as they comply with ICRU 50/62 safety margins and homogeneity requirements.

Normal tissue tolerance

DVH’s of both lungs, the heart and spinal cord will be obtained for all patients. Dose-Volume- Histograms (DVH’s) will mainly be used to document the normal tissue damage. DVH’s may also help to select the most appropriate treatment plan.

The risks for severe pneumonitis for patients treated under this protocol will be minimized as the volume of both lungs will be limited by the use of BEV planning and field-shaping (with optimal sparing of both lungs). The spinal cord tolerance (50 Gy) will not be exceeded with this technique.

External beam equipment

Radiation therapy will be delivered with megavoltage equipment with photon energies of equal to or greater than 6 MV. A multi-leaf collimator or individually shaped blocks will be used to shape the irradiation portal according to the planning target volume.

Dose specification

The prescription dose will be specified at the ICRU 50/62 reference point, which will be the isocenter for most patients. The daily prescription dose will be 1.8 Gy at the ICRU reference point and the 95% isodose must encompass the entire planning target volume (PTV). The maximum to the PTV must not exceed the prescription dose by >7% (ICRU 50/62 guidelines). Tissue density inhomogeneity correction will be used.

Treatment verification

Portal images will be obtained during the first fraction of all fields. On indication portal images will be repeated.

## Immunotherapy regimen

Pembrolizumab (ICI targeting PD-1) will be given concurrently on day 1 and 22 of the neoadjuvant therapy at a dose of 200 mg. For patients weighing < 50 kg, pembrolizumab will be given at a dose of 100 mg.

## Surgery

Surgery should be arranged 4 to 6 weeks after completion of PPCT. Each patient underwent either open or minimally invasive esophagectomy (McKeown or Ivor-Lewis esophagectomy utilizing video-assisted or robotic-assisted techniques), along with a 2-field or 3-field lymphadenectomy. Radical resection will be defined by both macroscopic observation and postoperative pathological negative margin. A jejunum tube will be placed via the nose or by jejunostomy.

The lymph nodes resected during total lymphadenectomy include the left recurrent laryngeal nerve nodes, right recurrent laryngeal nerve nodes, infraaortic arch nodes, periesophageal nodes of the upper, middle and lower thoracic portion, the infracarinal nodes, the posterior mediastinal nodes, the paracardiac nodes, lesser curvature nodes, the left gastric nodes, the common hepatic nodes, the splenic nodes and the celiac nodes.

For carcinomas proximal to the tracheal bifurcation a transthoracic esophageal resection is preferred. For carcinomas distal of the tracheal bifurcation but proximal to the gastro-esophageal junction, a transthoracic approach with a two-field lymph node dissection or a transhiatal approach can be performed, depending on both patient characteristics and local expertise. For distal tumors involving the gastro-esophageal junction a transhiatal esophageal resection is preferred.

A wide local excision including the N1 lymph nodes is carried out in both techniques including a standard excision of the lymph nodes around the coeliac axis (separately collected with left gastric artery marked by a suture). The continuity of the digestive tract will be restored by a gastric tube reconstruction or colonic interposition procedure with an anastomosis in the neck.

## Dose modifications / Retreatment delay

### Chemotherapeutic toxicity

For chemoradiotherapy-related AEs occurred during the neoadjuvant period, clinical observation, dose modification, suspension of the chemotherapy or radiotherapy, and symptomatic treatment can be applied according to the type and severity of the AEs and based on multidisciplinary discussion.

Hematologic Related Toxicity

If day 8, 15, 22, 29, and 36 the WBC are < 1.0 and/or platelets < 50: delay chemotherapy by 1 week until recovery above these values.

In case of febrile neutropenia (granulo < 0.5/L and fever > 38.5 ^o^C) or in case of severe bleeding or requiring ≥ 2 platelet transfusions, further chemotherapy will be withheld.

Non-hematologic Toxicity

These effects will be graded according to CTCAE recommendations for grading of acute and sub-acute toxicity.

Hypersensitivity Reactions

Hypersensitivity reactions will be classified as mild, moderate or severe. Definitions and

management guidelines are outlined below:

| **Classification of reactions** | **Management of reactions** |
| --- | --- |
| Mild symptoms  (e.g., mild flushing, rash, pruritis) | Complete infusion. Supervise at bedside. No treatment required. |
| Moderate symptoms (e.g., moderate rash, flushing mild dyspnea, chest discomfort, mild hypotension) | Stop infusion, give IV antihistamine (Clemastine 2 mg IV and Dexamethasone 10 mg IV), → after recovery of symptoms resume infusion at a rate of 20 ml/h for 15 minutes then 50 ml/h for 15 minutes then, if no further symptoms, at full dose rate until infusion is complete. |
| Severe symptoms (e.g., one or more of the following): respiratory distress requiring treatment, generalized urticaria, angioedema, hypotension requiring therapy) | Stop infusion, give IV antihistamine and steroid as above. Add epinephrine or bronchodilators if indicated, report as an adverse event, the patient will go off protocol therapy. |

Other toxic reaction and the prescribed management of these reactions are outlined in the following table.

| **Reaction** | **Management of reaction** |
| --- | --- |
| Renal  Creatinin ≤ 1.5 x the upper limit of normal at the day of retreatment.  Creatinin is > 1.5 x the upper limit of normal. | Continue therapy.  Establish intravenous infusion the evening preceding treatment at a rate to correct any volume deficits and produce a urine flow ≥ 50 ml/h.  Repeat serum creatinin value in the morning:  ≤ 1.5 x the upper limit of normal → Proceed treatment.  > 1.5 x the upper normal limit → Stop chemotherapy. |
| Gastrointestinal  Mucositis with oral ulcers or protracted vomiting despite antiemetic premedication. | Delay chemotherapy one week. |
| Neurologic  ≤ CTCAE grade 2.  CTCAE > grade 2. | Continue therapy.  Stop chemotherapy. |
| Cardiac  Asymptomatic bradycardia or isolated and asymptomatic ventricular extrasystoles. | Continue therapy under continuous cardiac  monitoring. |
| First degree AV block.  Symptomatic arrhythmia or AV block (excepts 1st degree) or other heart blocks. | Continue therapy under continuous cardiac monitoring.  Stop Paclitaxel/Nab-paclitaxel infusion, manage arrhythmia according to standard practice, patient goes off protocol. |
| Other Major Organ Toxicity  CTCAE grade > 2  (with the exception of esophagitis) | Stop therapy, patient goes off protocol treatment. |

### Radiation toxicity

Radiotherapy, especially concurrent with chemotherapy can lead to acute esophagitis. In some cases, medical support and/or a feeding tube will be necessary.

In the event of grade 4 radiation induced esophagitis both chemotherapy and radiotherapy will be withheld until the esophagitis recovered to grade 3.

Other acute complications of the radiation therapy are erythema, cough, nausea, fatigue and weight loss.

In the first weeks to six months after the irradiation radiation pneumonitis or fistula formation can occur.

### Immune-related toxicity

When facing immune-related AEs, dose modification of pembrolizumab is not recommended.

## Labeling

Labels will be prepared in accordance with Good Manufacturing Practice (GMP) and local regulatory guidelines. The label shall comply with the labeling requirements of Annex 13 of GMP. The label content will be translated into the local language.

## Storage

All investigational drugs should be stored in a safe place with appropriate storage conditions and should only be dispensed by a pharmacist or qualified designated person. The IP label on the package specifies the appropriate storage conditions.

## Drug inventory

The investigator will be responsible for inventorying all clinical supplies and maintaining records (release, inventory, and return) in accordance with the sponsor's instructions and in compliance with GCP guidelines and applicable international and/or national regulations. Subjects should be asked to return all unused medications and used medication packages at each visit to assess study medication adherence.

## Compliance

During the study period, study medication use and any deviation from the study protocol will be recorded in the eCRF, including the date and reason of medication use. The investigator shall explain to the subject the importance of receiving the investigational treatment as prescribed in the protocol and shall require the subject to take the medication as prescribed in the protocol.

# Study procedures

## Treatment plan

Pre-Treatment Evaluation and Evaluation on Therapy

| **Parameter** | **Pretreatment** | **Weekly during therapy** | **After completion of chemoradiotherapy** |
| --- | --- | --- | --- |
| **History, Physical** | ✘ | ✘ | ✘ |
| **Performance status** | ✘ | ✘ | ✘ |
| **Hematology** | ✘ | ✘ | ✘ |
| **Biochemistry** | ✘ | ✘ | ✘ |
| **Endoscopy** | ✘ |  |  |
| **EUS** | **If necessary** |  |  |
| **CT neck, thorax, abdomen** | ✘ |  | ✘ |
| **PET-CT** | ✘ |  |  |
| **Ultrasonography neck** | **If necessary** |  |  |
| **Pulmonary function test** | ✘ |  | ✘ |
| **ECG** | ✘ |  | ✘ |
| **Echocardiography** | ✘ |  | ✘ |
| **Toxicity** | **Baseline** | ✘ | ✘ |
| **Pregnancy test (if indicated)** | **Baseline** |  |  |
| **QLQ-C30, OES24** | ✘ |  | ✘ |

Table 1: Treatment Schedule

## Baseline evaluation

The evaluation includes physical examination, upper gastrointestinal endoscopy with tumor biopsy (if not previously examined), endoscopic ultrasonography (if necessary), contrast-enhanced neck, chest, and abdominal CT, PET-CT, routine blood test, echocardiography, pulmonary function, and electrocardiogram. Ultrasonography with fine-needle aspiration will be performed for any suspected cervical lymph node metastasis.

## Follow-up

The postoperative follow-up should be arranged in accordance with the National Comprehensive Cancer Network guideline for esophageal cancer.^18^

Follow-up visits will be scheduled at 1, 6, 12, 18, 24, 30, 36, 48, and 60 months after surgery. When patients are facing suspected local recurrence or distance metastasis, additional visit will be scheduled, and contrast-enhanced neck, chest, and abdominal CT, PET-CT, and/or upper gastrointestinal endoscopy will be performed.

The full-time study nurses will be responsible for the registration, follow-up of patients and inputting data of CRF. Then they will submit the information to the central office for further analysis. Each patient should have their own archive for evaluation (CRF), and a full-time study nurse will be responsible for the follow- up of each patient, as well as registration, inputting, and preservation of information.

## Patient discontinuation

Criteria for Discontinuing Treatment

- Patients may be removed from the protocol treatment in the following instances:

- Intercurrent illness, which would, in the judgment of the investigator, affects clinical status to a significant degree, and requires discontinuation of the chemotherapy and/or radiotherapy.

- Unacceptable toxicity (see paragraph 5.3.1).

- Request by the patient to withdraw.

- Completion of protocol treatment.

Therapy after completion of protocol treatment or discontinuation is at the investigator's

discretion.

## Effectiveness evaluation

**Pathological and Radiological Evaluation**

Each resected specimen will be evaluated by 2 pathologists independently. Pathological reports should describe tumor size, extent of tumor invasion, overall and positive lymph nodes dissected, resection margin, grade of differentiation, and grade of tumor regression. Pathological stage will be determined according to the American Joint Committee on Cancer criteria for esophageal carcinoma (eighth edition).^18^ R0 resection is predefined as a tumor-free resection margin. To evaluate and grade the response of esophageal tumor to PPCT, the extent of residual disease will be divided into 4 categories: grade I, no sign of residual carcinoma; grade II,50%.^4, 19^ Major pathologic response is predefined as no more than 10% of residual viable tumor cells in primary lesions.^20, 21^ During radiological evaluation of esophageal tumor, thickening of the esophageal wall observed in neck/chest/abdominal CT examination will be recorded as T3, and direct involvement of adjacent organs will be recorded as T4.^22^ The status of complete metabolic response will be used for radiological evaluation, which is predefined as maximum standardized uptake value.

**Pathology**

The resection specimen will be evaluated essentially using the standard protocol (margins, tumor type and extension, lymph nodes). The most recent UICC protocol is used for TNM classification and stage grouping (6-th edition, 2002).

In these resection specimens, special attention should be given to the effects of the preoperative chemoradiation, i.e., tumor reduction and therapy effects. It might be difficult to see tumor tissue macroscopically or tumor at all as a consequence of the chemoradiation. However, there is always a lesion, such as an ulcer or an irregular area covered by mucosa. This region plus surrounding areas need to be embedded in total in order to adequately judge 1) residual tumor and 2) therapy effects. In case of an adenocarcinoma the total distal esophagus plus gastro-esophageal junction should be embedded. The result of the chemoradiation varies from zero to 100%. This implies that one might see a unifocal vital tumor with no visible therapy effects or an area with therapy effects only, but no vital tumor cells. In many cases, a multifocal tumor appearance is present with intertwined therapy effects. In some cases, only scattered tumor cells are visible, often with bizarre morphologies. In these cases, a keratin stain can be helpful. Therapy effects include necrosis, inflammation with multinucleated giant cells, fibrosis and calcifications. In general fibrosis is the most remarkable effect, and it can be used to judge the extension of the untreated tumor.

The lymph node dissection should contain at least 10 nodes derived from both regional (mediastinal, esophageal) and distant sites (celiac region). The latter are important for correct TNM staging. Therapy effects can also be found in lymph nodes, e.g., as squamous remnants or mucin pools, but with absence of vital tumor cells. Deeper levels and a keratin stain can be helpful.

The resection margins, esp. the circumferential margin, should be evaluated with a 1mm cut-off point for vital tumor. This implies that the tumor-free margin should be >1mm. If vital tumor is present at ≤1mm from the surgical resection margin it should be considered positive.

The pathology report should contain the following: site of the tumor/lesion, type and grade of the tumor, extension into the esophageal wall, resection margins, therapy effects (with extension), lymph node status incl. the site, and the number of nodes with therapy effects.

## Security evaluation

Safety will be monitored by vital signs, physical examination, chest X-ray or CT examination, 12-lead ECG, laboratory tests, and all adverse events collected.

In addition to completing all planned safety assessments according to the frequency of visits specified in the protocol, the investigator may arrange planned visits according to the actual clinical conditions of the subjects.

# Concomitant therapy

Palliative and supportive care for disease related symptoms will be offered to all patients on this trial. Details of these will be collected on the case report forms.

Patients should not receive any other anti-cancer agents while on this protocol.

Patients will not receive prophylactically colony stimulating factors (e.g., G-CSF; GM-CSF) while on this study. However, in the event of protracted febrile neutropenia and life-threatening infection, such cytokines may be indicated. Thus, CSFs are not prohibited but their use must be recorded as concomitant medications on the case report forms.

# Data management

## Data collection

Designated investigator staff will enter the data required by the protocol into the electronic case report forms (eCRF). These persons will not be given access to the EDC (electronic data capture) system until they have been adequately trained. Automatic validation during data entry will check for data discrepancies and, by generating appropriate error messages, allow these data to be confirmed or corrected. The investigator must certify that the data entered into the eCRF are complete and accurate. After database lock, the investigator will receive a CD-ROM or paper copies of the patient data for archiving at the study site.

In case of technical problems, a paper CRF is available. All forms must be identified with patient pseudonym, date of the observation, and center number. Paper forms will be completed using a black ballpoint pen, and entries must be legible. Errors should be crossed by a single line but not obliterated, the correction inserted, and the change initialed and dated by the investigator or an authorized member of the study staff.

## Confidentiality

All patient-related data are recorded in a pseudonymized way. Each patient is unequivocally identified by a trial subject number, attributed at recruitment into the study. The investigator has to keep a patient identification log, including the full name and address of the subject and eventually additional relevant personal data.

## Investigator site file

Each participating trial site will file relevant documents (protocol, CVs, approvals of the authorities etc.) and trial related correspondence in the investigator site file (ISF).

# Statistical analysis

According to a review of current literature, the pCR rate after neoadjuvant chemoradiotherapy for locally advanced ESCC in Asian population was expected to be 43.2%.^23^ Meanwhile, the pCR rate after PPCT was assumed to be 56%, based on the short-term results of our prior PALACE-1 trial.^17^ With a power of 80%, a sample size of 130 will be required to detect a difference in pCR rate between PPCT and chemoradiotherapy at a significance level of 5%. To allow for a 10%of dropout cases, the sample size was increased to 143 in this PALACE-2 trial. To ensure the safety of the PALACE regimen, 20 patients were allocated to the phase 1 component, while 123 patients were assigned to the phase 2 component.

Subgroups will be defined according to pathological and radiological responses, baseline characteristics, long-term survival, and PD-L1 expression status. Data will be recorded and collected via standardized case report form and will be analyzed centrally.

# Assessment of response and toxicity

**Pathological response**

Comparisons will be drawn between pathological and, preoperative, clinical TNM stages to assess pathological responses.

**Toxicity**

Toxicity will be evaluated using the common toxicity criteria NCI-CTC criteria version 2 and Radiotherapy Oncology Group (RTOG) criteria (see appendix I + II). All patients will be evaluable for toxicity from the time of their first dose of Paclitaxel/Nab-paclitaxel and Carboplatin and the start of the radiotherapy.

# Ethical consideration

The responsible physician will inform the patient about the background and present knowledge on the drugs under study with special reference to known activity and toxicity. It must be emphasized that the patient is allowed to refuse the treatment either before or at any time during the study. Before the patient is entered in the study the patient's written consent will be obtained.

The principal investigator will ensure that this study will be carried out in agreement with either the "Declaration of Helsinki, Tokyo, Venice" or the laws and regulation of the country, whichever provides greater protection of the individual. The study will be approved by the institutional ethical review committee.

# Safey

## Definitions

### Adverse event

Adverse Event (AE) refers to all adverse medical events that occur after the subject has signed the informed consent form, which can be manifested as symptoms, signs, diseases, or abnormalities in laboratory tests, but may not necessarily have a causal relationship with the investigational drug.

### Serious adverse event

According to ICH, a Serious Adverse Event (SAE) is an adverse event that meets any of the following criteria:

• results in death

• is life-threatening: the patient is at a risk of death at the time of the event. It does not refer to an event which hypothetically might have caused death if it were more severe

• requires in-patient hospitalization or prolongation of existing hospitalization: hospital admissions and/or surgical operations planned before or during a study are not considered adverse events if the illness or disease existed before the patient was enrolled in the study, provided that it did not deteriorate in an unexpected way during the study

• results in persistent or significant disability/incapacity

• is a congenital abnormality/birth defect

• is a medically significant event, as judged by the Investigator

In contrast to routine safety assessments, SAE are monitored continuously.

## Collection of adverse events

Researchers and any designated person are responsible for detecting, archiving, and recording events that meet the AE or SAE definitions.

### Collection period

All adverse events (AEs) and serious adverse events (SAEs) will be collected throughout the study treatment period and follow-up from the signing of the informed consent form. If an event occurring after the safety follow-up period specified above is determined to be a late-onset toxicity of the investigational drug, it should be reported as an AE or SAE, as applicable.

### Collection method

Be careful not to introduce bias when detecting adverse events and/or serious adverse events. Open and unguided oral questioning of subjects is the preferred method of asking about the occurrence of adverse events. For example:

- "How are you feeling?"
- "Has your health improved or deteriorated since your last study visit?"

The investigator should observe all subjects for any abnormalities that occur during the clinical study, including adverse events, serious adverse events, drug combinations, clinical laboratory tests (routine blood, urine, blood biochemistry, coagulation function), 12-lead electrocardiogram, vital signs monitoring, physical examination, and unscheduled tests.

## Follow-up of Adverse Events

After the AE/SAE is first known, the investigator will need to actively follow up each subject at follow-up visits/contacts to obtain additional information. All serious adverse events, adverse events associated with the intervention study, or those that cause participants to discontinue the intervention should be followed until the event resolves (including return to baseline), long-term stable status changes, loss of follow-up, death, no further follow-up deemed necessary by the investigator, or other reasonable explanation. Follow-up can be selected according to the severity of adverse events in hospital, outpatient, home visit, telephone visit and other forms.

## Record and assessment of adverse event

### Record

The investigator is responsible for reviewing all documentation related to the event (e.g., hospital progress notes, laboratory tests, and diagnostic reports) and recording all relevant AE/SAE information in the case report form. The investigator will try to determine the diagnosis of each event based on signs, symptoms, and/or other clinical information. Where possible, the diagnosis (rather than individual signs/symptoms) is recorded as AE/SAE. If the diagnosis is unclear, the AE name can be temporarily reported as symptoms, signs, and abnormal examination, and each symptom, sign, and examination is recorded separately. When the diagnosis is clear, the records should be updated.

In the collection and evaluation process of AE and SAE, it is necessary to record the name of AE, start time, end time or outcome, severity, concomitant disease, co-medication, event description, causal relationship evaluation, and measures taken for the study drug due to adverse events.

### Assessment of severity

The severity grading of the adverse event according to the NCICTCAE V5.0 or if not applicable, the event will be graded as 1 = mild, 2 = moderate, 3 = severe, 4 = life-threatening.

### Assessment of causality

**Investigator’s assessment of the causal relationship of the adverse events to protocol treatment**

Assessment of Causal Relationship

Adverse events are assessed as not related, unlikely related, possibly related, probably related or definitely related to the study drug. The relationship to the protocol treatment of all adverse events will be categorized according to the following table:

| Criteria for Determining Category of Relationship of Clinical Adverse Events to Treatment | | |
| --- | --- | --- |
| 1 | **Not related** | This category applies to those adverse events which, after careful consideration, are clearly and incontrovertibly due to extraneous causes (disease, environment, etc.) |
| 2 | **Unlikely (must have two of four criteria)** | In general, this category can be considered applicable to those adverse events which, after careful medical consideration at the time they are evaluated, are judged to be unrelated to the protocol treatment. An adverse event may be considered unlikely if or when:  1. It does not follow a reasonable temporal sequence from administration of the protocol treatment.  2. It could readily have been produced by the patient's clinical state, environmental or toxic factors, or other modes of therapy administered to the patient.  3. It does not follow a known pattern of response to the protocol treatment.  4. It does not reappear or worsen when the protocol treatment is readministered. |
| 3 | **Possibly (must have two of four criteria)** | This category applies to those adverse events for which, after careful medical consideration at the time they are evaluated, a connection with the protocol treatment appears unlikely but cannot be ruled out with certainty. An adverse event may be considered possibly related if or when:  1. It follows a reasonable temporal sequence from administration of the protocol treatment.  2. It could not readily have been produced by the patient's clinical state, environmental or toxic factors, or other modes of therapy administered to the patient.  3. It follows a known pattern of response to the protocol treatment. |
| 4 | **Probably (must have three of four criteria)** | This category applies to those adverse events for which, after careful medical consideration at the time they are evaluated, are felt with a high degree of certainty to be related to the test drug. An adverse event may be considered probably related if or when: 1. It follows a reasonable temporal sequence from administration of the protocol treatment.  2. It could not be reasonably explained by the known characteristics of the patient's clinical state, environmental or toxic factors, or other modes of therapy administered to the patient.  3. It disappears or decreases on cessation or reduction in dose. There are important exceptions when an adverse event does not disappear upon discontinuation of the protocol treatment, yet drug-relatedness clearly exists (e.g. bone marrow depression, fixed drug eruptions, tardive dyskinesia).  4. It follows a known pattern of response to the protocol treatment. |
| 5 | **Definitely (must have all criteria)** | This category applies to those adverse events which, the Investigator feels are incontrovertibly related to protocol treatment. An adverse event may be assigned an attribution of definitely related if or when:  1. It follows a reasonable temporal sequence from administration of the protocol treatment.  2. It could not be reasonably explained by the known characteristics of the patient's clinical state, environmental or toxic factors, or other modes of therapy administered to the patient.  3. It disappears or decreases on cessation or reduction in dose with re-exposure to the protocol treatment. (Note: this is not to be construed as requiring re-exposure of the patient, however, a category of definitely related can only be used when a recurrence is observed.)  4. It follows a known pattern of response to the protocol treatment. |

### Outcome

The outcome of the adverse event according to the following definitions:

• recovered (adverse event disappeared)

• not yet recovered (adverse event is still existing, or patient is recovering)

• alive with sequelae (adverse event results in permanent disability/incapacity)

• death (patient died)

• unknown (only if patient was lost to follow up)

### Disease progression

Disease progression refers to the deterioration of a patient's condition as a result of the disease treated by the investigational drug. It may be an increase in the severity of the disease studied and/or an increase in the symptoms of the disease. The presence of new metastases or the progression of existing metastases in the primary tumor under study should be considered disease progression rather than AE or SAE. Events that are clearly due to disease progression during the study period should not be reported as AE.

### New tumors

New tumors should be classified as SAE. New primary tumors are not the primary reason for the use of the investigational drug and are found after participants are enrolled in the study.

### Death

All deaths that occurred during the study period or during the follow-up period after the final dosing specified in the study protocol must be reported as follows:

- Deaths determined to be due to disease progression shall be reported to the sponsor during the next monitoring visit and shall be recorded in the eCRF but not as SAE reports.
- When the death is not (or is not clearly) due to disease progression, the AE that caused the death must be reported within 24 hours as an SAE.
- Unexplained deaths should be reported as SAE. An autopsy may be helpful in assessing the cause of death. If an autopsy is performed, a copy of the autopsy report should be forwarded to the Sponsor pharmacovigilance team or its representative within the usual time frame.

A death occurring during treatment within 4 weeks after stopping treatment, whether treatment related or not must be reported to the investigator.

### AESI

Adverse event of special interest (AESI) is defined as an AE that requires special scientific and medical attention in order to understand the investigational product, may require close monitoring, and the investigator needs to inform the sponsor quickly. AESI can be severe or non-severe.

## Reports of adverse event

### Serious adverse event reporting requirements

Any unexpected clinical adverse event or abnormal laboratory test value that is serious, including death or overdose, occurring during the course of the study, irrespective of the treatment received by the patient, must be reported to the study coordinator within one working day of occurrence.

### Drug overdose

Drug overdose is defined as when a subject uses (intentionally or accidentally) more than the prescribed dose of a drug. If an overdose occurs, the subject may be given appropriate symptomatic and supportive treatment. Any adverse event due to drug overdose should be reported as an adverse event.

If an investigational drug overdose occurs during the course of the study, the investigator or other research Center personnel shall immediately inform the appropriate sponsor representative, no later than 24 hours after the overdose is known.

Drug overdoses combined with SAE are reported in accordance with SAE standard reporting methods and timelines.

## Pregnancy

In order to ensure the safety of subjects, for any pregnancy that occurs during the study period, the investigator or study Center staff must complete a paper pregnancy report form within 24 hours of learning of the pregnancy event and report it to the sponsor or sponsor pharmacovigilance representative. If the pregnancy is combined with SAE (abnormal pregnancy outcomes should be considered SAE), reporting should be done in accordance with SAE reporting deadlines and requirements. All pregnancy outcomes (spontaneous abortion, elective termination, ectopic pregnancy, normal delivery, or congenital malformation) should be followed up and documented, even after the participant has withdrawn from the study.

When pregnancy outcome information is available, the same reporting time limit should be observed.

# Entry and registration procedures

The name of the responsible physician and the institution will be recorded. The name of the patient will not be asked for nor recorded. A sequential identification number will be attributed to each patient registered in the trial. This number will identify the patient and must be included on all case report forms. In order to avoid identification errors, patient’s initials (maximum of 3 letters), date of birth and the name of the hospital will also be reported on the case report forms.

**Documentation**

All clinical observations must be entered into the appropriate part of the clinical record form by the investigator. The investigator must retain a list identifying patients to facilitate clarification of data by the study coordinators. In this study case report forms must be completed and submitted to the clinical study coordinator within one month after the assessment of response and the observation of progression, respectively, to allow accurate, timely analysis of the study results.

**Monitoring**

The study coordinator will ensure that key items of data transcribed onto the medical record forms, such as treatment dates, efficacy parameters and laboratory safety results, will be checked against source documents without compromising patient confidentiality, and any inconsistencies will be resolved.

# Address list

Department of Thoracic Surgery, Ruijin Hospital, Shanghai Jiao Tong University School of Medicine; 197 Ruijin 2nd Road, Shanghai 200025, China.

# Administrative and legal obligations

## Protocol amendments

Without the consent of the sponsor, review and approval of the EC, the researcher shall strictly abide by the current protocol and shall not change the protocol (ICH E6 Section 4.5.2).

Any modification to the protocol impacting on the conduct of the study, the potential benefit of the study, or which may affect patient safety, including changes of study objectives and endpoints, study design, patient population, sample sizes, study procedures, or significant administrative aspects will require a formal amendment to the protocol.

Any amendment to the protocol will be agreed upon by the lead coordinating investigator and the sponsor. Updated versions of the study documents will be submitted to the competent authority and the responsible ethical committee prior to implementation.

Administrative or technical changes of the protocol (minor corrections and/or clarifications) that do not affect study conduct, nor change the risk-benefit-assessment, will be agreed upon by the lead coordinating investigator and the sponsor and will be documented in a memorandum to the protocol. The responsible ethical committee will be informed about such changes at the discretion of the sponsor/lead coordinating investigator. The sponsor/lead coordinating investigator will assure that all documents related to protocol amendments have been included in the investigator site file at all participating study centers.

## Trial documentation and data storage

The investigator must ensure that all records pertaining to the conduct of the clinical study, including signed (e)CRFs, informed consent forms, drug accountability records, source documents, and other study documentation are adequately stored for the required time period to allow for review and reconstruction of the study. This documentation must be retained for 10 years following completion of the study or for the length of time requested by the sponsor.

## Trial termination

This clinical trial may be completed regularly as planned or may be prematurely discontinued by the sponsor. If the trial is completed regularly, the sponsor/sponsor representative will notify the competent authority as well as the competent ethics committee in writing. If the trial is prematurely terminated or suspended, the sponsor/sponsor representative will inform the competent authority as well as the competent ethics committee of the termination or suspension and the reason(s) for the termination or suspension within 15 days in accordance with national regulations.

# Publication and registration of the study

The results of this study will be published by the lead coordinating investigator (Hecheng Li) after final analysis has been performed. Publication will be independent of the nature of the results obtained (whether they were positive or negative). The manuscript written for publication, together with the materials provided by the statistician can be accepted as the final study report.

This clinical trial has been registered at a primary register of the WHO, at www.clinicaltrials.gov.

# References

1. Bray, F. *et al.* Global cancer statistics 2018: GLOBOCAN estimates of incidence and mortality worldwide for 36 cancers in 185 countries. *CA Cancer J Clin* **68**, 394-424 (2018).

2. Abnet, C.C., Arnold, M. & Wei, W.Q. Epidemiology of Esophageal Squamous Cell Carcinoma. *Gastroenterology* **154**, 360-373 (2018).

3. Shapiro, J. *et al.* Neoadjuvant chemoradiotherapy plus surgery versus surgery alone for oesophageal or junctional cancer (CROSS): long-term results of a randomised controlled trial. *Lancet Oncol* **16**, 1090-1098 (2015).

4. van Hagen, P. *et al.* Preoperative chemoradiotherapy for esophageal or junctional cancer. *N Engl J Med* **366**, 2074-2084 (2012).

5. Cancer Genome Atlas Research, N. *et al.* Integrated genomic characterization of oesophageal carcinoma. *Nature* **541**, 169-175 (2017).

6. Lin, D.C. *et al.* Genomic and molecular characterization of esophageal squamous cell carcinoma. *Nat Genet* **46**, 467-473 (2014).

7. Ohigashi, Y. *et al.* Clinical significance of programmed death-1 ligand-1 and programmed death-1 ligand-2 expression in human esophageal cancer. *Clin Cancer Res* **11**, 2947-2953 (2005).

8. Song, Y. *et al.* Identification of genomic alterations in oesophageal squamous cell cancer. *Nature* **509**, 91-95 (2014).

9. Zhang, L. *et al.* Genomic analyses reveal mutational signatures and frequently altered genes in esophageal squamous cell carcinoma. *Am J Hum Genet* **96**, 597-611 (2015).

10. Jardim, D.L., Goodman, A., de Melo Gagliato, D. & Kurzrock, R. The Challenges of Tumor Mutational Burden as an Immunotherapy Biomarker. *Cancer Cell* **39**, 154-173 (2021).

11. Sholl, L.M. *et al.* The Promises and Challenges of Tumor Mutation Burden as an Immunotherapy Biomarker: A Perspective from the International Association for the Study of Lung Cancer Pathology Committee. *J Thorac Oncol* **15**, 1409-1424 (2020).

12. Yarchoan, M., Johnson, B.A., 3rd, Lutz, E.R., Laheru, D.A. & Jaffee, E.M. Targeting neoantigens to augment antitumour immunity. *Nat Rev Cancer* **17**, 569 (2017).

13. Shah, M.A. *et al.* Efficacy and Safety of Pembrolizumab for Heavily Pretreated Patients With Advanced, Metastatic Adenocarcinoma or Squamous Cell Carcinoma of the Esophagus: The Phase 2 KEYNOTE-180 Study. *JAMA Oncol* **5**, 546-550 (2019).

14. Kojima, T. *et al.* Randomized Phase III KEYNOTE-181 Study of Pembrolizumab Versus Chemotherapy in Advanced Esophageal Cancer. *J Clin Oncol* **38**, 4138-4148 (2020).

15. Sharabi, A.B., Lim, M., DeWeese, T.L. & Drake, C.G. Radiation and checkpoint blockade immunotherapy: radiosensitisation and potential mechanisms of synergy. *Lancet Oncol* **16**, e498-509 (2015).

16. Sharma, P. & Allison, J.P. The future of immune checkpoint therapy. *Science* **348**, 56-61 (2015).

17. Li, C. *et al.* Preoperative pembrolizumab combined with chemoradiotherapy for oesophageal squamous cell carcinoma (PALACE-1). *Eur J Cancer* **144**, 232-241 (2021).

18. Ajani, J.A. *et al.* Esophageal and Esophagogastric Junction Cancers, Version 2.2019, NCCN Clinical Practice Guidelines in Oncology. *J Natl Compr Canc Netw* **17**, 855-883 (2019).

19. Chirieac, L.R. *et al.* Posttherapy pathologic stage predicts survival in patients with esophageal carcinoma receiving preoperative chemoradiation. *Cancer* **103**, 1347-1355 (2005).

20. Li, C.Y. *et al.* Predictors of Survival in Esophageal Squamous Cell Carcinoma with Pathologic Major Response after Neoadjuvant Chemoradiation Therapy and Surgery: The Impact of Chemotherapy Protocols. *Biomed Res Int* **2016**, 6423297 (2016).

21. Travis, W.D. *et al.* IASLC Multidisciplinary Recommendations for Pathologic Assessment of Lung Cancer Resection Specimens After Neoadjuvant Therapy. *J Thorac Oncol* **15**, 709-740 (2020).

22. Nishimaki, T. *et al.* Evaluation of the accuracy of preoperative staging in thoracic esophageal cancer. *Ann Thorac Surg* **68**, 2059-2064 (1999).

23. Yang, H. *et al.* Neoadjuvant Chemoradiotherapy Followed by Surgery Versus Surgery Alone for Locally Advanced Squamous Cell Carcinoma of the Esophagus (NEOCRTEC5010): A Phase III Multicenter, Randomized, Open-Label Clinical Trial. *J Clin Oncol* **36**, 2796-2803 (2018).
