# Supplementary material for: Preoperative pembrolizumab (anti-PD-1 antibody) combined with chemoradiotherapy for esophageal squamous cell carcinoma: a phase 1/2 trial (PALACE-2)
Source: Signal Transduct Target Ther. 2025 Nov 28;10:386. doi: 10.1038/s41392-025-02477-4 (PMC12660869; doi:10.1038/s41392-025-02477-4)
Supplement: Supplementary file 3 — Supplementary information [file 41392_2025_2477_MOESM3_ESM.docx]

**STATISTICAL ANALYSIS PLAN**

**Preoperative pembrolizumab combined with chemoradiotherapy for esophageal squamous cell carcinoma:**

**A multicenter phase 1/2 trial**

**(Abbreviated title: PALACE-2)**

Version / Date: V1.1 / August 2020

TABLE OF CONTENTS

[LIST OF ABBREVIATIONS 3](#_Toc180140952)

[1 Introduction 4](#_Toc180140953)

[1.1 Objective 4](#_Toc180140954)

[1.2 Study design 4](#_Toc180140955)

[1.3 Endpoints 4](#_Toc180140956)

[2 Statistical hypotheses 5](#_Toc180140957)

[3 Sample size estimation 5](#_Toc180140958)

[4 Randomization and allocation concealment 5](#_Toc180140959)

[5 Blinding 5](#_Toc180140960)

[6 Statistical analysis 6](#_Toc180140961)

[6.1 General principles 6](#_Toc180140962)

[6.2 Analyses of categorical variables 6](#_Toc180140963)

[6.3 Analyses of continuous variables 6](#_Toc180140964)

[6.4 Analyses of time-to-event data 6](#_Toc180140965)

[6.5 Missing data strategy 6](#_Toc180140966)

[6.6 Statistical analysis set 6](#_Toc180140967)

[6.7 Interim analysis 7](#_Toc180140968)

# LIST OF ABBREVIATIONS

| **Abbreviation/Term** | **Definition/Explanation** |
| --- | --- |
| AEs | adverse events |
| CI | confidence interval |
| DBL | database lock |
| DFS | disease-free survival |
| ESCC | esophageal squamous cell carcinoma |
| IQR | interquartile range |
| OS | overall survival |
| pCR | pathologic complete response |
| PPCT | preoperative pembrolizumab combined with chemoradiotherapy |
| SAP | statistical analysis plan |
| SD | standard deviation |

# Introduction

This statistical analysis plan (SAP) describes the statistical analysis methods and the form of result presentation regarding the Preoperative pembrolizumab combined with chemoradiotherapy for esophageal squamous cell carcinoma, a muti-center phase 1/2 study (PALACE-2). This SAP, including a comprehensive and detailed statistical strategy, rationale, and methodology for assessing the efficacy and safety, will be finalized and approved before database lock (DBL).

## Objective

This study aims to confirm the safety, feasibility and efficacy of preoperative pembrolizumab combined with chemoradiotherapy (PPCT) for esophageal squamous cell carcinoma (ESCC).

## Study design

The PALACE-2 trial is a multicenter, prospective, single-arm phase 1/2 clinical trial (ClinicalTrials.gov registration: NCT03792347 for phase 1, NCT04435197 for phase 2), examining the efficacy and safety of PPCT for locally advanced ESCC. Three medical centers in China are participating in this study (Ruijin Hospital, Shanghai Jiao Tong University School of Medicine; Cancer Hospital, Chinese Academy of Medical Sciences; and the First Affiliated Hospital of Nanchang University). Patients will receive PPCT, which includes concurrent pembrolizumab, carboplatin, paclitaxel (phase 1)/nab-paclitaxel (phase 2), and radiotherapy. Esophagectomy will be performed within 4 to 6 weeks after the completion of PPCT.

## Endpoints

| **Endpoints** | **Definition** |
| --- | --- |
| Primary endpoints |  |
| Pathologic complete response (pCR) rate | The percentage of patients who undergo surgery and achieve the absence of any signs of cancer in resected tissue samples examined by pathologists |
| Secondary endpoints |  |
| 3-year disease-free survival (DFS) rate | The percentage of patients who are still alive without any evidence of disease 3 years after esophagectomy |
| 3-year overall survival (OS) rate | The percentage of patients who are still alive 3 years after esophagectomy |
| R0 resection rate | The percentage of patients who undergo surgery and achieve a tumor-free resection margin |
| Rate of adverse events (AEs) during neoadjuvant therapy and perioperative period | The percentage of patients who have AEs during neoadjuvant therapy and perioperative period.  AEs will be evaluated and recorded according to the National Cancer Institute Common Terminology Criteria for Adverse Events (version 5.0). |

# Statistical hypotheses

Our hypothesis is that patients who receive PPCT will achieve a better pCR rate than patients after neoadjuvant chemoradiotherapy.

# Sample size estimation

According to a review of current literature, the pCR rate after neoadjuvant chemoradiotherapy for locally advanced ESCC in Asian population was expected to be 43.2%. Meanwhile, the pCR rate after PPCT was assumed to be 56%, based on the short-term results of our prior PALACE-1 trial. With a power of 80%, a sample size of 130 will be required to detect a difference in pCR rate between PPCT and chemoradiotherapy at a significance level of 5%. To allow for a 10%of dropout cases, the sample size was increased to 143 in this PALACE-2 trial. To ensure the safety of the PALACE regimen, 20 patients were allocated to the phase I component, while 123 patients were assigned to the phase II component.

# Randomization and allocation concealment

This is a single-arm trial, so randomization is not applicable.

# Blinding

This is an open-label trial, so blinding is not applicable.

# Statistical analysis

## General principles

All statistical analyses will be performed using SPSS 22.0 (IBM), R (version3.5.3, R Foundation for Statistical Computing, Vienna, Austria) or GraphPad Prism version 8.0.0 for Windows (GraphPad Software). The significance level for testing differences will be set at α=0.05 (bilateral). A significance level of P < 0.05 will be considered statistically significant, and a 95% confidence interval (CI) will be used.

## Analyses of categorical variables

Categorical variables will be summarized by descriptive statistics, including the number of participants, number of events, and percentage. We will use Pearson Chi-squared test or Fisher exact test to compare the difference between the two groups.

## Analyses of continuous variables

Continuous variables following a normal distribution will be presented as mean ± standard deviation (SD), and the Student’s t-test or one-way ANOVA test will be used for comparison. In cases of noncompliance with the normal distribution, continuous variables will be presented as medians [interquartile range, (IQR)] and compared using the Wilcoxon rank-sum test.

## Analyses of time-to-event data

Regarding the survival data, the Kaplan-Meier approach will be used to calculate OS and DFS at 1, 2, 3, 4, and 5 year(s). The estimates of median survival time will be provided.

## Missing data strategy

Missing data will not be imputed.

## Statistical analysis set

The per-protocol population will be defined as all patients who received PPCT and esophagectomy. Perioperative AEs, short-term efficacy and postoperative survival will be analyzed in the per-protocol population. Patients who received PPCT will be included in the analysis for neoadjuvant toxicity.

## Interim analysis

No scheduled interim analysis will be conducted in this study.
